# More Than 1,001 Problems with Protein Domain Databases: Transmembrane Regions, Signal Peptides and the Issue of Sequence Homology

**DOI:** 10.1371/journal.pcbi.1000867

**Published:** 2010-07-29

**Authors:** Wing-Cheong Wong, Sebastian Maurer-Stroh, Frank Eisenhaber

**Affiliations:** 1 Bioinformatics Institute (BII), Agency for Science, Technology and Research (A*STAR), Singapore; 2 School of Biological Sciences (SBS), Nanyang Technological University (NTU), Singapore; 3 Department of Biological Sciences (DBS), National University of Singapore (NUS), Singapore; 4 School of Computer Engineering (SCE), Nanyang Technological University (NTU), Singapore; University of California San Diego, United States of America

## Abstract

Large-scale genome sequencing gained general importance for life science because functional annotation of otherwise experimentally uncharacterized sequences is made possible by the theory of biomolecular sequence homology. Historically, the paradigm of similarity of protein sequences implying common structure, function and ancestry was generalized based on studies of globular domains. Having the same fold imposes strict conditions over the packing in the hydrophobic core requiring similarity of hydrophobic patterns. The implications of sequence similarity among non-globular protein segments have not been studied to the same extent; nevertheless, homology considerations are silently extended for them. This appears especially detrimental in the case of transmembrane helices (TMs) and signal peptides (SPs) where sequence similarity is necessarily a consequence of physical requirements rather than common ancestry. Thus, matching of SPs/TMs creates the illusion of matching hydrophobic cores. Therefore, inclusion of SPs/TMs into domain models can give rise to wrong annotations. More than 1001 domains among the 10,340 models of Pfam release 23 and 18 domains of SMART version 6 (out of 809) contain SP/TM regions. As expected, fragment-mode HMM searches generate promiscuous hits limited to solely the SP/TM part among clearly unrelated proteins. More worryingly, we show explicit examples that the scores of clearly false-positive hits, even in global-mode searches, can be elevated into the significance range just by matching the hydrophobic runs. In the PIR iProClass database v3.74 using conservative criteria, we find that at least between 2.1% and 13.6% of its annotated Pfam hits appear unjustified for a set of validated domain models. Thus, false-positive domain hits enforced by SP/TM regions can lead to dramatic annotation errors where the hit has nothing in common with the problematic domain model except the SP/TM region itself. We suggest a workflow of flagging problematic hits arising from SP/TM-containing models for critical reconsideration by annotation users.

## Author Summary

Sequence homology is a fundamental principle of biology. It implies common phylogenetic ancestry of genes and, subsequently, similarity of their protein products with regard to amino acid sequence, three-dimensional structure and molecular and cellular function. Originally an esoteric concept, homology with the proxy of sequence similarity is used to justify the transfer of functional annotation from well-studied protein examples to new sequences. Yet, functional annotation via sequence similarity seems to have hit a plateau in recent years since relentless annotation transfer led to error propagation across sequence databases; thus, leading experimental follow-up work astray. It must be emphasized that the trinity of sequence, 3D structural and functional similarity has only been proven for globular segments of proteins. For non-globular regions, similarity of sequence is not necessarily a result of divergent evolution from a common ancestor but the consequence of amino acid sequence bias. In our investigation, we found that protein domain databases contain many domain models with transmembrane regions and signal peptides, non-globular segments of proteins having hydrophobic bias. Many proteins have inherited completely wrong function assignments from these domain models. We fear that future function predictions will turn out futile if this issue is not immediately addressed.

## Introduction

Following the request of a collaborator to hypothesize about the function of Eco1, an uncharacterized yeast gene at that time, the application of the full battery of sequence-based prediction tools [1], [2] revealed an apparently significant hit to the Pfam domain PF00583 [3] in the local search mode Figure S1). This finding helped to identify a potential acetyl-CoA binding site and, subsequently, the hypothesis of Eco1's acetyltransferase function was proven experimentally [4]. At about the same time, another collaborator inquired about the function of the protein “Alt a 1” of the fungus *Alternaria alternata* (AAB40400). The same approach revealed an apparently significant hit to the Pfam domain PF00497 (Figure S2) indicating some relationship to bacterial extracellular solute-binding proteins. The initial hope of having found at least something to follow up faded away quickly when it became clear that the query has just a signal peptide (SP) in common with the proteins belonging to domain PF00497. This SP has artificially elevated the alignment score into the significance range and, thus, created the impression of functional relatedness. Why do the domain models perform so differently?

The theory of biomolecular sequence homology and its practical application for predicting function for uncharacterized genes by annotation transfer from well-studied homologues is one of the few achievements of theoretical biology that have significance for all fields of life science [5], [6]. Similarity of amino acid sequences implies, to a certain degree, similarity in 3D structure and biological function [7]–[9]. Even apparently unrelated sequences with essentially zero sequence identity can adopt the same structural fold. This fact is rationalized by the conservation of the seemingly random, intricate hydrophobic pattern in the amino acid sequence of globular proteins that is required to form the tightly packed hydrophobic core of the tertiary structure [10]. This level of statistically significant sequence similarity is thought to arise from common ancestry under the pressure of selection at each step of mutational divergence with only rare instances of convergent evolution [11], [12]. The corresponding evolutionarily favored amino acid exchanges tend to maintain side chain hydrophobicity, charge and side chain volume. Not surprisingly, it is these exchanges that score highly in the BLOSUM62 matrix [13] used in the BLAST/PSI-BLAST suite [14], [15].

This general theme has received two variations. The first is introduced by the notion of the protein domain [16]–[18] and the existence of multi-domain proteins. Structurally, domains are protein sequence segments that form their own 3D structure with its independent hydrophobic core (and with a generally more polar surface); thermodynamically, they fold and melt independently; from the evolutionary point of view, these sequence segment are shuffled in the genome as independent units and are re-used in different contexts [5]. With respect to the homology search, the notion of domains leads to segmentation of protein sequences where the segments represent homologous members of a sequence family with the same type of domain. The family collection can become laborious; thus, protein domain libraries have appeared as a collective effort of the scientific community. Among the collections, there are PROSITE [19], BLOCKS [20], PRINTS [21], SUPERFAMILY [22], CDD [23], TIGRFAM [24], Panther [25], ProDom [26], EVEREST [27], the libraries of Y. Wolf and L. Aravind published with IMPALA [28] and, as the most systematically developed primary collections, Pfam [3] and SMART (Simple Modular Architecture Research Tool [29]).

The second issue is that many segments do not have globular structures at all [30]–[32]. They can be of fibrillar nature, transmembrane (TM) helices, disordered regions, etc. Typically, these regions have a clear amino acid compositional bias or a primitive repetitive pattern. Sequence similarity between two sequence segments of this type does not necessarily mean common ancestry but is obviously an enforced result of physico-chemical constraints. For example, long hydrophobic stretches such as transmembrane helices appear similar regardless of ancestry and, as in the introductory example, all signal peptides [33] but also GPI lipid anchor sites [34], [35] or coiled coil regions [36] must look alike to a certain degree. Many types of polar non-globular regions, for example serine-rich segments, readily compensate for insertions/deletions or substitutions as long as the integral properties of the respective subsegments remain unchanged. Consequently, convergent evolution might have a more significant role for non-globular sequences.

Thus, sequence similarity can either be due to homology (common ancestry) or convergent evolution (common selective pressure). The criterion of sequence similarity for inferring homology is actually applicable only to globular segments and non-globular parts should be excluded from starting sequences in homology searches. The special case with amino acid compositional bias was recognized early and it was always advised to exclude those segments from similarity searches when hunting after distantly related proteins. For the BLAST/PSI-BLAST suite, the SEG program was advised to suppress at least the most obvious low complexity regions [14], [37] besides the application of statistical corrections for compositional bias [37], [38]. Sequence family searching heuristics should consider excluding also other types of non-globular segments such as coiled coil regions from the similarity search [39]. In the original concept of SMART [40], special care was paid to determine domain boundaries correctly, to include all secondary structural elements of globules, for example by matching the alignment section with known 3D structures, and to exclude all sequence parts such as polar or proline-rich linker regions that do not belong to the domain considered.

The unsupervised inclusion of transmembrane helices and signal peptide segments in homology searches is especially prone to erroneous addition of unrelated sequences to the sequence family under study since the systematic coincidence of hydrophobic positions creates the appearance of similarity in the hydrophobic pattern, otherwise the key to sequence homology among globular sequence segments [10]. The consequently generated high similarity score as in the introductory example of “Alt a 1” might support an otherwise unjustified annotation transfer and lead to wrongly predicted function if it were not detected by manual checks.

Similar precautions are generally out of scope when protein domain model libraries are applied for function prediction over query sequences, especially in a genome-wide mode. It is desirable to have systematic factors that might cause spurious annotations such as isolated similarities to signal peptides or some types of transmembrane helices be suppressed during the annotation workflow.

When checking domain databases for the inclusion of transmembrane helices and signal peptides into the domain model, we found more than thousand domain instances in Pfam and a couple of examples even in SMART. These hidden Markov models (HMMs) can be a systematic cause of spurious similarity hits especially if the HMM-based sequence scan is applied in the local search mode. In this work, we wish to emphasize that these domain models can also give rise to wrong hits even in the global search mode where the high score from the membrane-helical part can mask the absence of match for the associated globular domains. For support of the reader, database search results, alignments, domain library entry lists and files with “cleanup” domain models as referred to in the following text are provided as supplementary material at the associated WWW site http://mendel.bii.a-star.edu.sg/SEQUENCES/ProblemDomains-TMSP/.

## Results

### Search for transmembrane helices and signal peptides included in SMART database alignments and validation of findings

Since the SMART database [29] is relatively small and the alignments are very well curated, its alignments were used as a test ground for a SP/TM detection algorithm as described in detail in the Methods section.

In brief, we recovered the full length protein sequences that contained the segments in a given alignment of SMART version 6, applied 5 TM and 2 SP predictors published in the literature and we checked overlap of predicted SP/TM regions with the alignment segments. For an alignment position to be considered part of a predicted TM or SP region, the respective residue must be included into the predicted range in a critical number of sequences and by a certain number of prediction tools determined by a statistical criterion based on the binomial distribution (significance value 0.05).

For each predicted TM or SP region, we derive a score as the arithmetic mean of the logarithmic probabilities of SP/TM prediction over all alignment columns involved (Methods, equation 5). The false-positive prediction rate was assessed using the SCOP α-helical proteins and the SCOP membrane class (Structural Classification of Proteins [41], [42]) to determine TM- and SP-score cutoffs with false-positive rates below 5%.

In contrast to the Pfam test described below, SMART version 6 alignments contain pleasantly few SP/TM regions. With a TM-score cutoff of ≥−12 (FP rate of 4.67%) and SP-score cutoff of ≥−1 (FP rate of 4.02%), the number of predicted TM helices and signal peptides are 40 and 5 respectively. At the domain level, this translates to 13 problematic domains with TMs and 5 with SPs, respectively (Table 1). Thus, the fraction of problematic domains is very low with 1.6% (13/809) having TMs and 0.6% (5/809) SPs segments.

**Table 1:** Summary of predicted/validated non-globular segments and supporting evidence for the 18 SMART version 6 domains.

Domain name	Type	Predicted segments	Validated Segments	Comments
SM00019 : SF_P (Pulmonary surfactant protein)	TM	33–58	1–58^#^	The N-terminal propeptide 1–58 of NP_003009 forms a TM when induced by a Brichos domain [99].
SM00157 : PRP (Major prion protein)	TM	117–140	112–135^#^	Latent transmembrane region in human prion protein BAG32277 [100], [101].
SM00665 : B561 (Cytochrome B561/ferric reductase TM domain)	TM	4–146	N/a	Intrinsic membrane protein [102].
SM00714 : LITAF (LPS-induced tumor necrosis factor α factor)	TM	38–61	N/a	The LITAF domain appears to have a membrane-inserted motif (although without transmembrane segment) [103].
SM00724 : TLC (TRAM, LAG1 and CLN8 homology domains)	TM	10–76; 216–238; 287–307	N/a	Proof for 8 membrane-spanning segments in Lag1p (NP_011860) and Lac1p (NP_012917) [104]
SM00730 : PSN (Presenilin, signal peptide peptidase, family)	TM	5–27; 113–134; 214–285; 600–649	4–25^#^; 115–133^#^; 214–231^#^; 241–257^#^; 260–283^#^; 602–621^#^; 628–644^#^	Out of 10 TM regions shown for human presenilin-1 (AAB46371), 9 are in the domain alignment out of which 7 are predicted here [105].
SM00752 : HTTM Horizontally transferred transmembrane domain	TM	12–25; 75–95; 275–294; 338–357	N/a	Domain is known to have 4 TM regions [80].
SM00756 : VKc (catalytic subunit of vitamin K epoxide reductase)	TM	12–30; 104–192	13–32^#^; 142–189^#^	VKORC1 (Q9BQB6) is a membrane protein [106].
SM00780 : PIG-X (Mammalian PIG-X and yeast PBN1)	TM	230–248	230–252^#^	PBN1 (CAA42392) is a type I transmembrane protein in the endoplasmic reticulum [107].
SM00786 : SHR3_chaperone (ER membrane protein SH3)	TM	7–111; 167–186	N/a	Shr3p (NP_010069) has 4 membrane segments [108].
SM00793 : AgrB (Accessory gene regulator B)	TM	42–204	N/a	*S. aureus* ABW06464 is a membrane protein [109].
SM00815 : AMA-1 (Apical membrane antigen 1)	TM	522–527	515–602^#^	Segment missing in structure 1W81_A [110].
SM00831 : Cation_ATPase_N (Cation transporter/ATPase, N-terminus)	TM	72–90	65–94^#^	Segment maps to a TM helix of the ß-domain of 1KJU_A [111].
SM00190 : IL4_13 (Interleukin 4/13)	SP	1–20	1–23^#^	Annotated as secreted. Segment missing in structure 1ITL_A [112].
SM00476 : DNaseIc (deoxyribonuclease I)	SP	1–19	1–17^#^	Annotated as secreted. Segment missing in structure 1DNK_A [113].
SM00770 : Zn_dep_PLPC (Zn-dependent phospholipase C, α toxin)	SP	4–26	1–64^#^	Annotated as secreted. Segment missing in structure 1OLP_A [114].
SM00792 : Agouti	SP	1–19	1–89^#^	Annotated as secreted. Segment missing in structure 1Y7J_A [115].
SM00817 : Amelin (Ameloblastin precursor)	SP	11–28	1–26^#^	Protein AAG27036 [116] is secreted to enamel matrix.

Both the predicted and, if explicitly available in the literature, the validated segments of TM regions or signal peptides are provided in the sequence count of the respective SMART domain alignment. In cases marked with “#”, the sequence positions are with respect to the reference sequence given in the comments.

These 18 predictions were manually validated: (i) If the respective predicted segments were indeed structural helices and not SPs/TMs, they should be part of one of the nearest globular domains in the sequence. The alignment sequences were searched against the sequences with known 3D structure from the Protein Data Bank (PDB) for any significant hits (with the generous Blast E-value≤0.1) and we checked whether the predicted SP/TM region overlaps with the segment covered by the structure. If the predicted SP/TM region was missing in the structure or if it was described as a TM helix in the structural report, we considered the prediction as validated. (ii) Without structural hits, we searched the scientific literature for topological information about membrane embedding of reference sequence segments.

**Figure 1:**
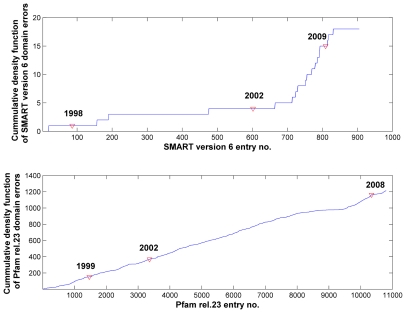
Cumulative plots of SMART version 6 and Pfam release 23 problematic domains. In SMART version 6, the total number of domains with predicted SP/TM segments peaks at 18, which made up 2.2% of 809 SMART domains (see top). Red triangles mark time points for the years 1998, 2002 and 2009 when the total number of domain models was 86, 600 and 809 respectively. In Pfam, the total number of problematic domains peaks at 1214, which made up 11.8% of 10340 Pfam domains (see bottom). Likewise, red triangles marked the years 1999, 2002 and 2008 with 1465, 3360 and 10340 Pfam entries respectively.

As the information collated in Table 1 confirms, none of the 18 cases is a false-positive SP/TM prediction. Thus, we conclude that the SMART domain database contains at least 18 problematic domain models. It is of interest to note that, except for 4 cases with accessions below SM00600, all other problematic domains have been added to SMART only in recent years (Figure 1).

### Detection of more than a thousand domains in the Pfam database with SP/TM regions

**Figure 2:**
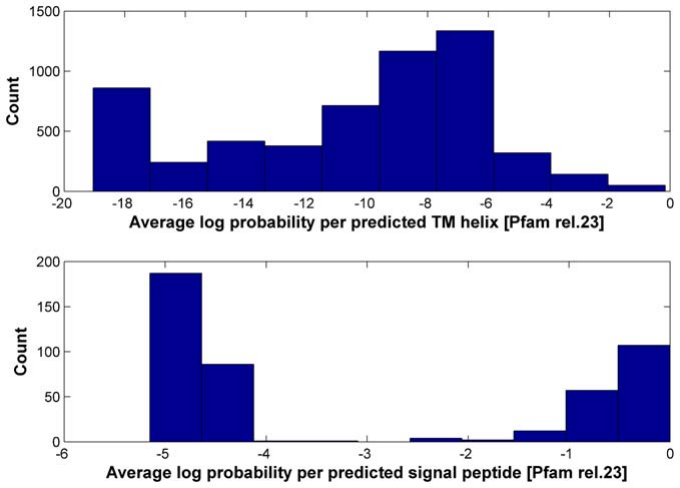
Histograms of average log probability per predicted transmembrane helix and per predicted signal peptide in Pfam release 23. The top part shows the histogram of average log probability per predicted transmembrane helix; the bottom part shows the same per predicted signal peptide. The log probability provided on the x-axis is calculated with equations 5 and 6. At the *TMcutoff* of ≥−12 (false-positive rate 4.67%) and *SPcutoff* of ≥−1 (false-positive rate 4.02%), the number of predicted TM helices and signal peptides are 3849 and 164 respectively.

Given that our SP/TM detection procedure provides statistical error measures for the prediction, it can be reasonably applied on the body of Pfam domain models. When this work was started, the available Pfam version was release 23 constructed with the HMMER2 package. About 19% (1937 out of 10340) of Pfam-A domains in release 23 [3], [43]–[45] do not have more than 4 seed sequences in the alignment and, consequently, there is not enough statistical power for rejecting the null hypotheses even if the predicted SP/TMs are true (see Methods). In Figure 2, we show the distributions of the TM- and SP-scores per predicted SP/TM region for the alignments of the remaining 8403 domains of Pfam-A. Both histograms exhibit a bimodal distribution where true-positives cluster at high scores and false-positive predictions aggregate at low scores (see Methods). If we apply the same SP/TM-score cutoffs as in the SMART exercise (−12 and −1 respectively), the number of predicted TM helices and signal peptides are 3849 and 164 respectively.

**Figure 3:**
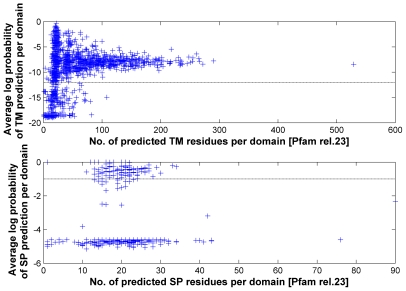
Average log probability plot of transmembrane helix and signal peptide predictions per domain. The top part shows the average log probability per predicted transmembrane helix calculated per domain; the bottom part shows the same per predicted signal peptide. Whereas the y-axis shows the log probability in accordance with equation 6 applied over all predicted segments for a given domain, the x-axis represents their cumulative length. At the *TMcutoff* of ≥−12 and *SPcutoff* of ≥−1 (horizontal dashed lines), the number of problematic TM and SP domains are 1079 and 164 respectively. The total number of problematic domains is 1214 (1050 TM, 135 SP and 29 concurrent TM and SP).

At the domain level, this implies 1079 (10.4%) and 164 (1.6%) out of 10340 Pfam-A domains having TM or SP regions included into the domain alignment (Figure 3). The extent of the non-globular part introduced by TM regions together with the polar linkers between them in the domain alignments of Pfam can be huge (more than 500 positions). Whereas SMART strived for excluding non-globular parts from the domain alignments and included a few critical domains only recently, this has not been a matter for Pfam at all (Figure 1). The accumulation of problematic domains was even over all the history of Pfam. Interestingly, our conservative estimate of 10.4% (1079/10340) for TM-containing Pfam-A domains measures well against the estimated 16.7% (1365/8183) for Pfam-A release 19 reported by Bernsel *et al.* [46] who just applied TMHMM. It should be noted that their result is from a plain application of TMHMM without any additional false-positive hit suppression.

Among our 164 domains with SP predictions, we might expect 6.6 (∼7) wrong predictions. On average, each domain with predicted TM regions contains about 3.6 (3849/1079) TM helices, out of which 0.17 (4.67% of 3.6) represent false-positive TM helices. We might expect that about 50 domains out of the 1079 domains are wrongly included into this list. Even if we remove those values from the total number of 1214 problematic domain models (1050 TM, 135 SP and 29 concurrent TM and SP errors), Pfam-A release 23 still contains more than 1001 critical cases as claimed in the title of this article.

### Inclusion of non-globular sequences leads to false-positives in homology searches, a serious source of errors in protein function annotation

The domain alignments in Pfam and SMART are used for the derivation of hidden Markov models (HMMs) that, in turn, are applied for searching matches in query sequences with programs of the HMMER packages [47]–[49] with HMMER2 being the currently validated version. It should be noted that both the local and the global search modes for domain hits are available.

**Figure 4:**
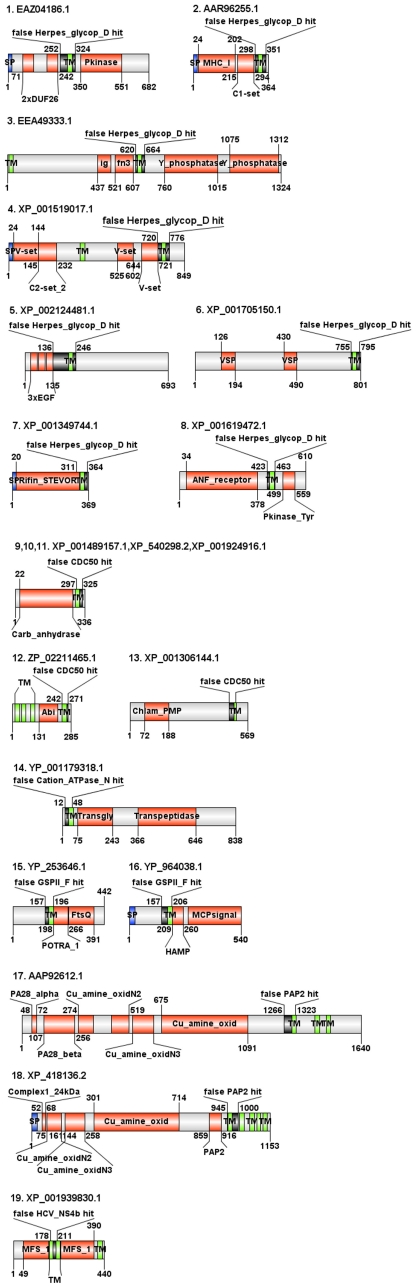
Examples of domain architectures of false-positive HMM hits caused by TM helices in the fragment-mode search. We show illustrative examples for six Pfam release 23 models: Herpes_glycop_D (PF01537.9), CDC50 (PF03381.7), Cation_ATPase_N (PF00690.18), GSPII_F (PF00482.11), PAP2 (PF01569.13) and HCV_NS4b (PF01001.11). The black boxes denote the problematic domain annotations in the respective sequences. Additional material such as hmmpfam outputs and alignments are available at the associated BII WWW site for this work. Domain architecture illustrations were created with DOG 1.5 [98].

With SP/TM regions as part of the domain alignment, the respective HMMs are no longer useful for local mode searches since a match in the TM or SP region alone without any other sequence similarity to the query sequence can be sufficient to cause a false-positive fragmentary domain hit as in the introductory case of “Alt a 1”. Further illustrative examples are provided in Table S1 and Figure 4. We especially searched for sequence examples having both hits with a SP/TM region containing domain model (with an alignment restricted to the SP/TM region only) as well as multiple other prediction tool hits that provide intrinsic annotation contradictions. Thus, we have two arguments supporting the idea that the SP/TM region containing hit is false-positive.

One of the referees brought up the argument that some of the sequences in Table S1 (and also in the subsequent Table S2) have become obsolete. In the revised Tables S1 and S2, we show that none of the sequence examples have disappeared; instead, the sequence entries have been updated and, in none of the cases of sequence edition, the computation results have been changed to the extent of compromising the conclusion. It needs to be emphasized that sequence-based prediction tools should be applicable to all types of sequences including naturally occurring ones, mutated versions, synthetic constructs as well as all types of hypothetical sequences. It is this ability of protein sequence analysis that makes it so powerful to conclude from genome sequences. For example, it should be noted that, sometimes, the absence of a domain hit is taken as indication of a sequence representing a non-coding RNA.

The model Herpes_glycop_D (PF01537.9) has a membrane-helix region that, together with its linkers on both side, are the sole part of a match in the fragmented search mode for a large variety of taxonomically and functionally diverse proteins out of which eight architectures are presented here. Similarly, the TM region (plus surrounding polar linkers) of model CDC50 (PF03381.7) significantly hits proteins with at least three different architectures in the fragmented HMM search.

For another 4 domain models Cation_ATPase_N (PF00690.1), GSPII_F (PF00482.11), PAP2 (PF01569.13) and HCV_NS4b (PF01001.11) provided as further illustration examples, the respective TM region hit a single TM helix segment of several seemingly unrelated proteins. In all cases, their alignment scores were above their family-wise gathering score thresholds.

Not surprisingly, the global search mode that forces a complete match of the domain model over a subsegment of the query sequence is the standard regime for running hmmsearch and hmmpfam of the HMMER2 package. Typically, a positive hit is recognized either by a score above a so-called gathering threshold (which is supplied together with and determined empirically by the creator of the Pfam domain model) or an E-value below a trusted limit (such as 0.1, see page 23 of the HMMER2 user guide). It is particularly worrying that a number of domain models with SP/TM regions included generate quite convincing E-values for unrelated sequences even in the global search mode. In all these cases, matches of a hydrophobic region in the query with the hydrophobic segments of these validated SP/TM regions is the reason for the elevated score that frequently surpasses even the gathering score threshold.

**Figure 5:**
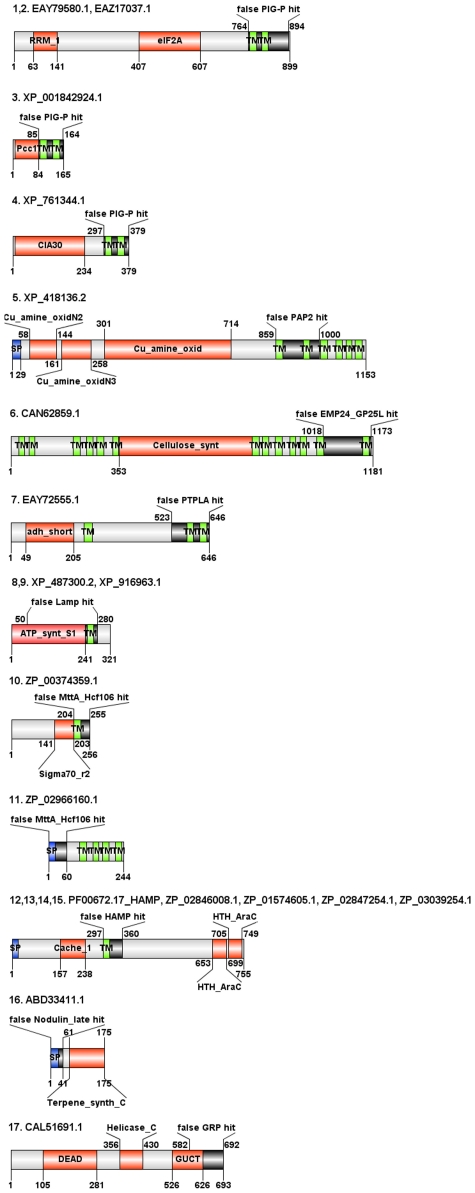
Examples of domain architectures of false-positive HMM hits caused by TM helices/signal peptdes in the global-mode search. Findings for nine Pfam release 23 models Pig-P (PF08510.4), PAP2(PF 01569.13), EMP24_GP25L (PF01105.15), PTPLA (PF04387.6), Lamp (PF01299.9), MttA_Hcf106 (PF02416.8), HAMP (PF00672.17), Nodulin_late (PF07127.3) and GRP (PF07172.3) are shown. The black boxes denote the problematic domain annotations in the respective sequences. Additional material such as hmmpfam outputs and alignments are available at the associated BII WWW site for this work. Domain architecture illustrations were created with DOG 1.5 [98].

To investigate the effects of SP/TM regions in homology searches, two separate HMM searches against the NR database were performed for each domain under study. The first run relied on an HMM using the original alignment. For the second run, we constructed a “cleanup” alignment via the removal of the predicted TM or SP segments. The two HMMs for the hmmls style of search (global with respect to the domain and local to the query sequence) were built from the alignments using the commands ‘hmmbuild –F –amino model-file alignment-file’ and ‘hmmcalibrate –seed 0 –num 5000’. When contrasting the results of the two HMM runs at E-value≤0.1, we assume all hits of the cleanup model as true-positives and scrutinize all additional hits of the original model as potential false-positive hits. We screened them for potentially contradictory annotation using sequence-analytic tools [1], [2] and scientific literature. Below, we describe several representative cases (Table S2, Figure 5).

The model PIG-P (PF08510.4) includes a segment with TM helices (positions 1–91) and hydrophilic region (positions 92–208). In the global-mode search against the non-redundant database, the first 100 alignment positions of the model (i.e., the N-terminal part with the 2 TM helices) hit a pair of C-terminal TM helices in the four protein targets listed in Table S2 (Figure 5). The positions of the HMM covering the cytoplasmic part of Pig-P [50] correspond mostly to a single large gap in the alignment with any of the four hit sequences (and this gap has only a marginal influence on the total score). The E-values both with the HMMER2 and HMMER3 suites are very convincing (between e-27 and e-09) and the scores are all far above the gathering threshold. Nevertheless, these are certainly false-positive hits. Whereas, the Pig-Ps are endoplasmic reticulum proteins [50], EAY79580.1, EAZ17037.1 and XP_001842924.1 have nucleic acid binding domains and are most likely nuclear proteins and XP_761344.1 appears mitochondrial due to a CIA30 domain [51]. Just having two TMs and their short linker matching is a poor argument for common ancestry.

The PAP2 (type 2 phosphatidic acid phosphatase) domain model (PF 01569.13) hits the sequence XP_418136.2 (Table S2, Figure 5) in an internal segment. Inspection of the alignment shows that the only high scoring similarity regions belong to the two transmembrane segments and there are two large gaps corresponding to non-membrane segments in PAP2 proteins. Most importantly, the two motifs A and C characteristic for PAP2 proteins are not conserved and the motif B is completely absent in the sequence hit [52]. Thus, this is a false-positive finding regardless of impressive scores and E-values.

The members of the EMP24_GP25L family (PF01105.15, Table S2) have a polar region, a coiled coil segment followed by a transmembrane part in their model [53]. Sequence CAN62859.1 (Figure 5) generates a significant, yet false-positive hit to the respective HMM although it does not have any traces of a heptade repeat in the sequence.

In the model for PTPLA (PF04387.6, Table S2), the first 30 N-terminal positions of the HMM contain the active site motif that is critical for function and, thus, for family membership [54], [55]. The alignment of EAY72555.1 (Figure 5) with the respective HMM has a large gap in this region; nevertheless, the matches with two transmembrane conveniently shift the E-value into the region of statistical significance although, this time, the score is below the gathering threshold.

The Lamp domain (PF01299.9, Table S2) characteristic of lysosomal glycoproteins hits sequences XP_487300.2 and XP_916963.1 of ATP synthases (Figure 5) significantly both with regard to score and E-value. Inspection of the alignments shows that a segment of about 120 HMM positions out of 340 is absent in the sequence hits since the respective region is covered by three large gaps. As a result, several critical functional motifs (cysteines 1–5 and the cytoplasmic tail GY motif [56]) are missing in the hits. The total score is rescued by the transmembrane region match.

The typical architecture of MttA_Hcf106 (PF02416.8, Table S2) proteins (known to be involved in sec-independent translocation [57]) comprises of a TM segment followed by an amphipathic helix and an acidic domain. The alignment of the respective HMM with the false-positive hit sequences ZP_00374359.1, an RNA polymerase, and ZP_02966160.1 (Figure 5), a putative phosphatase, shows good match in the TM segment (in the case of ZP_02966160.1, with its signal peptide !!) followed by a moderate fitting to the amphipathic helix segment and an almost complete absence of the acidic part.

HAMP protein segments comprise of two α-helices connected with a linker having a characteristic motif [58]. In addition, the domain model PF00672.17 (Table S2) includes a preceding transmembrane segment which causes significant, yet false-positive global search HMM hits in four proteins (see Table S2, Figure 5) although none of them has traces of the linker region (covered by a gap in the alignment).

The architecture of the Nodulin_late domain (PF07127.3, Table S2) consists of a signal peptide followed by a region with two characteristic cysteine pairs [59]. The protein ABD33411.1 is annotated as a nodulin_late protein in the database and, indeed, the respective HMM produces a significant hit by any commonly used statistical criterion; yet, the hit is false-positive (Figure 5) since the alignment is good only in the signal peptide region but this match is followed by two large gaps and none of the cysteine pairs is conserved.

Further, the domain model GRP (PF07172.3, Table S2) for cell-wall related proteins comprises of an N-terminal signal peptide followed by a glycine-rich region [60]. The respective HMM matches the C-terminal part of CAL51691.1, a putative RNA helicase (Figure 5). Surprisingly, the signal peptide part of the GRP domain matches the C-terminal two secondary structural elements of the GUCT domain (PF08152.4) in CAL51691.1 (a β-strand and an α-helix in the homologous structure 2E29 chain A [61]).

Our final example illustrates the issue with multiple TM segments. If the linkers between them differ among query and model, the gap penalties offset some part of the score accumulated by the hydrophobic position matches. The case of claudin proteins, small membrane glycoproteins with 4 TM helices and a length below 200 AA, is instructive in this respect. In a global search mode with the PMP22_claudin model (PF00822.12), the respective HMM hits numerous sequences of γ-subunits of voltage-dependent Ca-ion channels with E-values in the order of e-7. Closer inspection of the seed alignment showed that just a single channel sequence (CCG2_mouse) was included although they are not related to the family [62]. If we remove this entry, the new HMM still hits to 4TM γ-subunits of voltage-dependent Ca-ion channels (e.g., NP_542375.1, NP_542424.1) as well as to the 3TM XP_533601.2 (Natural killer cell protein) with E-values in the order of 0.08. In all cases, the sequence similarity is confined to matches of the hydrophobic segments.

### Inclusion of non-globular sequences leads to false-negatives in homology searches thus decreases sensitivity of the domain model

The decrease in specificity of domain models harboring SP/TM regions is also accompanied by a decrease in sensitivity. In general, the need to have additional good alignment scores for the SP/TM pieces can become a burden for any true-positive sequences that are incompletely sequenced or missing the SP/TM-region pieces naturally.

By contrasting the HMM runs between the original and cleanup models, potential false-negatives were identified as hits that were found only by the cleanup models. Then (see Methods), we re-computed the scores/E-values for the original HMM as well as another set of scores/E-values using the same HMM and EVD parameters from the original model but without the SP/TM segments (cleanup case). Finally, the two sets of scores/E-values were compared to find hits where their original score/E-values were less significant than their re-computed ones (i.e. without SP/TM). These were considered as false-negatives.

In Table S3, we show selected false-negative examples of several domain models with validated SP/TM-regions where their re-computed scores/E-values drastically improved without their SP/TM segment scores. All re-computed hits' scores except for NP_848488.2 were clearly above their gathering score thresholds. Previously, all these hits would be treated as false-positives if gathering score thresholds were considered. In essence, the negative scores of the SP/TM segments (due to their absence in the corresponding sequence) had acted as heavy penalties on the total scores, thus, it was concluded that these hits were insignificant.

### Significant rates of problematic function annotations in existing sequence databases due to SP/TM regions in domain models

It was already suggested in the literature that unsupervised annotation transfer based on spurious sequence similarities has created a myriad of false function annotations for sequences from genome projects [63]–[65]. If care is not exercised, the inclusion of SP/TM regions into domain models can become a perfect recipe for protein annotation disaster.

We explored this issue for PIR (Protein Identification Resource) iProClass v3.74 [66] and retrieved sequences with Pfam accession IDs for the problematic domains in Table 2. These sequences were re-annotated using HMMER2 hmmpfam in global-search mode (with parameter –null2). Interestingly, a number of sequences returned zero hmmpfam hits (searched for with a very permissive E-value ≤10) despite being annotated with the respective domains in the database and these are clearly false annotations (column 5). For each sequence with reproduced hit, summing up the match, insert and state transition log-odd scores (provided in the Pfam model) over its emitted HMM sequence allowed us to recalculate its total score as well as the SP/TM-region- (column 2) and non-SP/TM-segment-specific parts of the score log odd scores. We tagged a sequence as a potential false-positive hit if the total score was at least the gathering score threshold 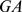 while its non-SP/TM-segment-specific score contribution was less than the expected non-SP/TM specific gathering score threshold 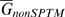 (column 4, see Methods); thus, only the match to the SP/TM hydrophobic region carries the hit over the threshold. Surprisingly, the number of unjustified annotations is between 2.1 to 13.6% depending on the type of domain (column 6); thus, the annotation error due to spurious SP/TM matches can be quite substantial.

**Table 2:** Unjustified annotation percentage of validated problematic domains in protein information resource (PIR) iproclass v3.74 (Global-mode search).

Domain Name	Type, validated region of model (size)	No. of retrieved sequences	No. of FP hits where 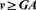 , 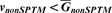	No. of annotations without hmmpfam hits (E>10)	Total No. of unjustified hits (%)
PF00690.18 : Cation_ATPase_N (Cation transporter/ATPase, N-terminus), 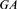 = 18.90, 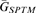 = 9.58, 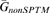 = 18.79, 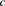 = −9.47, 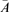 = −76.19	TM,66–87 (87), ref.[111]	3684	74	3	77 (2.1%)
PF01105.15 : EMP24_GP25L (Endoplasmic reticulum and golgi apparatus trafficking proteins), 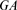 = −16.00, 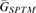 = 13.82, 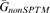 = −20.28, 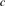 = −9.54, 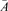 = −208.58	TM,141–167 (167), ref. [53]	1029	8	33	41 (4.0%)
PF01299.9 : Lamp (Lysosome-associated membrane glycoprotein), 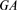 = −87, 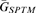 = 18.34, 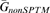 = −95.80, 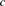 = −9.54, 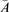 = −614.95	TM,304–340 (340), ref. [56]	164	2	12	14 (8.5%)
PF01544.10 : CorA (CorA-like Mg2+ transporter protein) 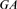 = −61.3, 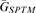 = 28.57, 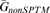 = −80.17, 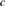 = −9.70, 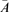 = −503.57	TM,341–407 (407), ref. [117]	2717	15	71	86 (3.2%)
PF01569.13 : PAP2 (type 2 phosphatidic acid phosphatase) 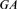 = 8.3, 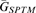 = 21.70, 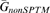 = −3.92, 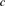 = −9.47, 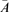 = −120.86	TM,102–177 (177), ref. [52]	5231	108	19	127 (2.4%)
PF02416.8 : MttA_Hcf106 (sec-independent translocation mechanism protein) 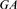 = 7, 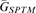 = 17.88, 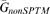 = −1.30, 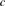 = −9.58, 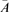 = −102.29	TM,1–19 (74), refs. [57], [118]	2085	283	0	283 (13.6%)
PF04387.6 : PTPLA (protein tyrosine phosphatase-like protein), 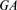 = 25, 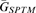 = 13.59, 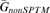 = 20.97, 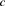 = −9.56, 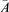 = −291.27	TM,89–168 (168), refs. [54], [55]	277	3	3	6 (2.2%)
PF04612.4 : Gsp_M (General secretion pathway, M protein) 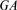 = 25, 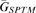 = 24.68, 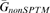 = 10.16, 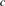 = −9.85, 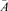 = −247.83	TM,1–40 (165), ref. [119]	401	19	6	25 (6.2%)
PF07127.3 : GRP (plant glycine rich proteins) 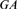 = 17.2, 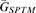 = 14.64, 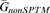 = 12.16, 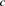 = −9.59, 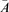 = −173.44	SP,1–49 (134), ref. [60]	207	12	4	16 (7.7%)
PF08294.3 : TIM21 (Mitochondrial import protein), 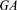 = −20.3, 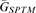 = 0.19, 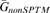 = −10.88, 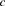 = −9.61, 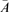 = −309.20	TM,1–36 (157), ref. [120]	118	7	1	8 (6.8%)
PF08510.4 : PIG-P (phosphatidylinositol N-acetyl-glucosaminyl transferase subunit P), 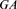 = −11.4, 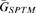 = 40.20, 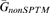 = −42.07, 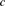 = −9.53, 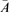 = −233.36	TM,1–67 (153), ref. [50]	143	4	0	4 (2.8%)

In the first column, we list selected Pfam domains with their accession, identifier, description and their gathering score (as in Pfam release 23) that have TM and/or SP regions included into the model. The region in the domain alignment that includes the validated SP/TM segments (together with interlinking loops as described in Methods) and the corresponding references are provided in the second column. The number of retrieved sequences from iProClass v3.74 with respect to each domain is given in the third column. The number of unjustified hits that returns results (and also satisfied the criteria) and without results are given in the next two columns. The last column gives the total and percentage of the unjustified hits with respect to the number of retrieved sequences. In addition, the log odd scores were re-derived from the match/insert/state transition scores provided by the respective HMM model. The reproduced scores 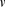 varied from the original scores at 0.57±0.34. 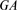 and 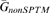 (see equations 19 and 20) denote the domain gathering score threshold and the expected non-SP/TM-specific gathering score threshold respectively.

Additional material such as hmmpfam outputs and alignments are available at the associated BII WWW site for this work.

### Sequence complexity of SP/TM-regions

The fact that signal peptide or transmembrane helix segments are of lower sequence complexity than their globular counterparts is not widespread general knowledge. To our current understanding, there is only a comment about this issue in the BAliBASE article of Bahr *et al.* [67] where the notion is considered “self-evident” without provision of any supporting data.

In brief, we extracted all sequences from Uni-Prot (release 14.4) with the feature keys “signal” and “transmem”. Among the single-transmembrane proteins, we selected those characterized as “anchor” in a special group. For multi-TM region proteins, we selected those who have 5–9 annotated TM segments. Additionally, we got the experimentally verified α-helical TM regions as provided by TMPDB (release 6.3) [68]. As a reference point for helices in globular proteins, we took the set of alpha-helices in PDB (extracted from PDBFIND2.txt as of April 2010 [69]) with 14–28 amino acid residue length surrounded by coil regions. Within all sets, sequence redundancy was suppressed with Cd-hit and a 50% sequence identity threshold [70].

In our calculations, we find that only 3% of residues in α-helices in globular domains are covered by hits of the quite stringent low complexity tool SEG (parameters window 12, 2.2, 2.5) [71] whereas this is the case for 18% for all residues in transmembrane helices extracted from TMPDB. Similarly, 24% of residues in signal peptides in UniProt are hit by the same SEG tool. Thus, SP and TM regions are more likely to be of low complexity than structural helices of comparable length.

Interestingly, the values for the Uni-Prot sets are 30% for single transmembrane proteins, 33% for single transmembrane proteins with the region annotated as “anchor” but only 12% for multi-transmembrane proteins. Thus, the problems with non-relevant matches in hydrophobic regions are more likely to occur, as a trend, in proteins having signal peptides or only a few transmembrane segments compared with cases of multi-membrane-spanning proteins.

## Discussion

### The notion of domain and the issue of SP/TM regions

There is no substitute for computational methods in large-scale functional annotation of sequence data and sequence similarity as surrogate for homology has to remain a decisive factor for function assignment [72]. E-value guided extrapolation of protein domain annotation has been a cornerstone for understanding completely sequenced genomes. There is about a decade of experience of using HMMER2 with a Pfam release 23-style or SMART domain library. These tools have indeed had tremendously high impact and have done a very good job.

The fundamental consideration in this article, namely the difficulty to interpret sequence similarity as a result of similarity of non-globular segments, (especially signal peptides or transmembrane regions) within the current theory of sequence homology, the basis of annotation transfer, goes beyond the specific criticism for a few domain models. In this context, it appears necessary to recall what the notion of a protein domain implies. In the introduction of their article, Veretnik *et.al.* [18] provide a list of definitions extracted from the literature and applicable in a variety of research contexts. The criteria involve sequence or 3D structure similarity, structural compactness, assignment and atomicity of associated biological function; yet, not any conserved piece of sequence can be considered a domain.

In the special case of globular domains that have tertiary structure, sequence similarities imply sequence homology as well as fold and function similarity. If 3D structures are known, domains as compact (having an own hydrophobic core) and spatially distinct units of protein structures that share significant structural similarity can be grouped together (for example, in libraries such as SCOP [41], [42] or CATH [73]). Structural domains are also units for folding and, in the thermodynamic sense, for melting [16]. It should be noted that, even for globular domains, sequence similarity does not guarantee the same structure and function, especially with sequence identities below 25% [7], [8], [74]. Whereas fold similarity is usually a consequence of hydrophobic pattern similarity, nevertheless, lots of the structural detail can be different affecting issues of conformational flexibility, binding specificity, catalytic activity, substrate preferences and, thus, biological function [1], [5].

Although structure-based domain libraries aim at providing complete and well-defined annotation about a domain, the antecedent of requiring structural information and associated function makes it exclusive for only a small number of well-studied proteins. Thus, many more proteins in sequence databases remain difficult to characterize under this definition.

Meanwhile, a complementary domain definition based on the sequence homology also evolved independently. In the sequence-analytic context, domains as the basic components of proteins are families of sequence segments of minimal length (i) that are similar to each other with statistical significance, (ii) that provide for a specific biological function at the molecular level (“atom” of molecular function [5]) and (iii) that occur in different sequence domain contexts as they are reshuffled by evolution [75]–[77]. Indeed, this notion is the basic to the approach of sequence homology-based domain libraries like SMART and Pfam. Yet, there is a caveat: Because of the statistical significance criterion, similarity between sequences to be established requires them to be without any type of amino acid compositional bias or primitive repetitive pattern. This condition essentially brings together the structural and the sequence-analytic definition of domain since both, essentially, become applicable only to the globular domain type. The exclusion of sequential bias makes the application of the sequence homology theory to non-globular sequence segments (in contrast to globular segments) at least a borderline case and, often (certainly at low sequence identity), disables sequence similarity as argument for common ancestry, similarity of structure (if there is any 3D structure at all) and function.

It is crucial to note that similarity of sequences can either be due to homology (common ancestry) or convergent evolution (common selective pressure due to physical requirements or biological function). We wish to emphasize that generally applied sequence-statistical criteria for deducing homology have been derived from studies of globular domains. In these cases, conservation of an intricate, only apparently random hydrophobic pattern is necessary for composing the hydrophobic core and, thus, for fold conservation [1], [10].

This condition is generally not fulfilled for non-globular segments (e.g., transmembrane helices, signal peptides, inter-domain linker regions, segments carrying lipid-attachment sites, etc.); thus, their functional annotation requires other methods than just annotation transfer based on position-wise sequence similarity. It appears likely that many types of non-globular segments re-occurring in evolutionary very distant proteins are rather the result of convergent evolution than common ancestry; for example, the likelihood of a *de novo* appearance of a phosphorylation site in a generally serine-rich stretch seems quite high in evolutionary time scales. This issue would deserve a more explicit study on its own.

In a generalized theme, SP/TM segments are usually the results of physico-chemical constraints and do not confer the specific biological function of the protein. Therefore, missing alignments in the SP/TM regions is less detrimental than that of the non-SP/TM regions if the membrane-embedded region is just used as translocation signal.

### About the suitability of HMM-type models to infer homology from SP/TM-region containing sequences

To further the argument, in the framework of HMM, there is no clear demarcation of SP/TM and non-SP/TM regions towards the computation of the alignment scores. Hence, this questions the correctness of inclusion of SP/TM regions into the HMM or, at least, makes a separate consideration for them a matter of necessity in the context of the homology argument.

Our arguments raise the question whether position-specific scoring matrices (PSSM), HMMs or profiles are indeed the appropriate tool to classify all kinds of non-globular segments with regard to sequence homology. Matching the hydrophobic pattern alone is recognized insufficient for inferring homology among proteins with transmembrane helices. In previous reports [46], [78], sequence similarity was attempted to be complemented with topology requirements. Anantharaman and Aravind [79] in their discussion with the reviewer list further arguments such as conservation of functional residue patterns, conservation of the number of TMs, the linker length, etc. Similar arguments are provided by Schultz [80]. If common ancestry is not a necessary requirement, PSSMs or HMMs are useful to test aspects of sequence similarity in context of physical pattern constraints (for example, as in the case of TMHMM [81] for the purpose of transmembrane helix prediction).

The case of SPs/TMs is of special importance since their hydrophobic stretches can create the false appearance of similarity to the respective hydrophobic core of the target template based on a hydrophobic pattern match. Alignments with many hydrophobic residues in the same columns generate high scores; thus, a SP/TM match can elevate an otherwise mediocre HMM score into the range of significance. The inclusion of a SP/TM into the domain model can compromise the selectivity of HMMs towards specific families and create hits not only to neighboring sequence families within the superfamily but also beyond. Whereas errors of the first kind might be considered not dramatic, we show with examples in Tables S1 and S2 that, most importantly, drastic cases of misannotation can happen.

Thus, the reliability in homology inference is greatly influenced by the amount of non-globular content in such domain library entries. We find that, even in the very well curated SMART domain collection (version 6), there are 18 domain models (out of 809) that include TMs or SPs. Based on our conservative approach, we find that clearly more than 1000 domains harbor SP/TM segments in Pfam release 23 (out of 10340 entries). To make matters worse, we observe a growing trend of addition of SP/TM region-containing domain models in Pfam and especially in SMART during the recent years (Figure 1).

In the Results section, we provide convincing examples that these domains have the potential to lead to annotation problems. They do not only cause promiscuous hits in fragment-mode HMM searches (Table S1). As we could see, the problems persist in the global-mode HMM searches by elevating the hits to significant levels beyond any normally applied E-value cutoffs or gathering score thresholds for a variety of SP/TM-region containing domain models (Table S2).

Therefore, our finding might suggest the mandatory removal of SPs/TMs from domain models. We do not recommend this at this stage. Such a strategy is not easy to implement due to several reasons. The required editing of domain libraries given their current status would be quite laborious and appears impractical in the short term. Then, there is also the issue with some multi-TM region protein domain models where there is little or no soluble globular component. Further, the biological significance of sequence similarity of proteins with TM regions and its relationship to homology has been studied only in a few cases [79], [80], [82], [83].

Notably with regard to signal peptides, the Pfam team has conveyed to us the removal of signal peptides in most domain models for future releases (Alex Bateman, personal communication). Similarly, it appears reasonable to remove TM regions from models where they are not integral parts of the globular domain and, especially, where the domain occurs also outside the TM region context. An excellent match between SP/TM regions of non-relevant proteins is possible just because of their uniform hydrophobicity and this match will elevate scores in alignments. Often, this might be insufficient to overcome thresholds of significance but, as we see in our experience, it can happen and it happens systematically for some types of models. Most likely, the problems arise with domains having one or very few TM regions which are the majority of cases in Pfam (366 with 1 TM helix, 170 with 2 TM helices, 127 with 3 TM helices, 416 with more than 4 TM helices as with our conservative estimates). As we have seen, the trend to low sequence complexity is especially strong for proteins segments representing a signal peptide or a single-TM anchor. Both the exclusion of signal peptides and of transmembrane helix anchors from domain models would remove the bulk (but not all) of the problems described in this article. Among all SP/TM regions, signal peptides, signal anchors and single TM regions have a trend to considerably more pronounced sequence complexity than TM regions in multi-TM proteins (see Results).

In addition, we propose two other possible workarounds: First, one might process each query sequence with tools recognizing non-globular segments including those for SP/TM regions and mask them with X-runs before comparing the query with domain libraries. Yet, this would not exclude cases such as SPs in HMMs hitting structural helices (see the GRP example CAL51691.1 from Table S2). Alternatively, we offer a supplementary, “cleanup” version of Pfam release 23 (see the file “Pfam_rel23_globalHMM_cleanup.rar” at the WWW site for this article). In cases of problematic domain models with SPs/TMs, hits of query sequences both with the original HMM as well as with the HMM derived from the reduced alignment without the respective SPs/TMs are to be compared. We suggest considering collinear hits of both models as benign whereas hits from only the original HMM should be flagged as problematic pending manual check by the user of the annotation. For this purpose, we supply versions of the domain model that are cleaned from transmembrane helical and signal peptide inclusions (see associated WWW site for this work).

Whereas this work explores the issue of SPs/TMs in domain models mainly based on an analysis of HMMER2 and Pfam release 23, both have concurrently been updated to HMMER3 and Pfam release 24 [84]. We wish to underline that this revision does not resolve the problems described in this paper. For 16 out of the 17 sequence examples provided in Table S2, using HMMER3 with Pfam release 24 produces the same false-positive hits. In the remaining case of CAL51691.1 and domain model GRP (PF07172), the alignment of the respective domain entry has not changed and the absence of hit appears due to an increased gathering score (17.2 for global-mode and 15.9 for fragment-mode HMMER2-search in Pfam release 23 in contrast with 22.7 for HMMER3 and Pfam release 24). We do not think that the transition to HMMER3 resolves the problem of SPs/TMs included into seed alignments since SPs/TMs will contribute to the score similarly to buried structural helices regardless of any composition-based corrections. On the contrary, we have seen that the fragment-mode search with HMMER2 has essentially been useless in the E-value guided mode because of many false hits; for the current HMMER3 beta-release, this is the only search mode available so far.

### E-value guided domain search *versus* gathering threshold criteria

As a remedy, switching from the E-value guided hit finding to gathering score thresholds is proposed. This is problematic from several viewpoints. The HMM concept has the beauty of a rigorous probabilistic formulation that allows a natural treatment for substitutions and gaps in the same formalized framework. Further, the introduction of E-values provides a handle to compare various types of predictions that hit the same sequence region. Unfortunately, the gathering score concept (an expert-defined domain-specific score threshold for homologous hit selection) brings in an arbitrary component into the prediction process.

Firstly, the determination of a gathering score is not guided by a fundamental consideration but, instead, depends on the data and literature situation at the time of seed alignment collection. Regardless, how carefully a gathering score is selected by the expert, it remains a subjective decision. The sequence with a true model hit with lowest score (as well as the false hit with the highest score) critically depends on the size of the non-redundant protein database, the variety of sequences therein and the quality of the seed alignment at the time of model construction. Sequence databases have a strong growth due to increasingly cheaper sequencing. With time, our biological knowledge grows and we know more about previously uncharacterized sequences. Not surprisingly, gathering thresholds have an inherent trend to be increased with time even if the underlying seed alignments do not change.

For example in the case of PF00583 (Acetyltransferase) in the introductory Eco1 example, the gathering scores have evolved the following way: Pfam5 (1999) with 6.5 (global mode/gm) and 6.5 (fragment- mode/fm), Pfam6 (2000) with 15 (gm) and 15 (fm) (with some shortening of the alignment compared with Pfam5), Pfam7 (2001) with 18.2 (gm) and 16.3(fm). The reader is invited to return to Figure S1 to verify that only the *Drosophila melanogaster* sequence AE003559 would make it over the gathering score threshold in 2000 and later whereas the Pfam5 gathering score would clearly support many homologues. Thus, the experimentally verified discovery of the Eco1 acetyltransferase might have been overseen after 2000 based on a gathering score criterion but it would never disappear from the radar in an E-value guided search at any time point. As for the other introductory example, the PF00497 (SBP_bac_3) model, the fragment-mode gathering thresholds have also been heavily changed over time: For Pfam5 (1999) and Pfam6 (2000) GA = −20 (fm), whereas, for Pfam7 (2001), GA = 49.9 (fm); thus, the sequence “Alt a 1” would have been a hit based on the gathering threshold criterion until the year 2000 but it would be suppressed with the more recent versions of Pfam. At the same time, the E-value generated by the example did not change.

Secondly, gathering scores hide the problem of balance between true-negative and false-positive hits. Although increasing gathering scores (as there is a trend in Pfam releases) reduce false-positive hit rates, this approach excludes a growing number of true hits and, thus, also limits the extrapolation power of domain models into the space of uncharacterized sequences. On the contrary, an E-value gives insights into the orders of magnitude of error rates when assuming the annotation transfer to be correct. The user of a gathering threshold guided assignment does have the illusion of dealing with ultimately correct hits; in contrast, an E-value provides a quantitative and typically non-zero statistical measure for annotation error.

Thirdly, gathering thresholds do not relate well with the statistics of hit distribution in the non-redundant database. In the HMMER2 manual, Sean Eddy says on page 22 “Calibrated HMMER E-values tend to be relatively accurate. E-values of 0.1 or less are, in general, significant hits”. Further on page 43, he writes “The best criterion of statistical significance is the E-value. The E-value is calculated from the bit score. It tells you how many false positives you would have expected to see at or above this bit score. Therefore a low E-value is best; an E-value of 0.1, for instance, means that there's only a 10% chance that you would've seen a hit this good in a search of non-homologous sequences. *Typically, I trust the results of HMMER searches at about E = 0.1 and below, and I examine the hits manually down to E = 10 or so.*”

**Figure 6:**
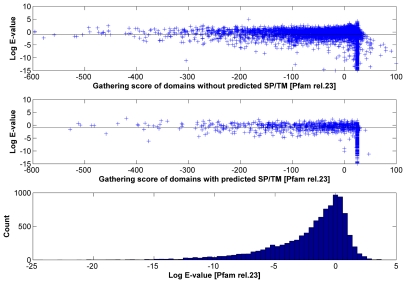
Relationship between the gathering score and the corresponding E-value threshold for Pfam domain library release 23. Whereas the y-axis shows the gathering score threshold (GA) for the global-mode search, x-axis shows the corresponding E-value threshold (in decimal log scale) calculated with the domain-specific extreme-value function with parameters provided in the corresponding HMM file (for an NR database size of 7365651 sequences) for this score. The upper plot represents the distribution for 9126 domains without detected SP/TM region, the middle part shows the same for the 1214 domains with SP/TM problems. Effectively, there is no clear correlation between gathering score and E-value threshold. If E-values close to 0.1 are considered significant, all dots should be close to the “−1” line (horizontal dashed lines) in this graph and, indeed, there is some agglomeration of data points in that area; yet, there are numerous outliers. Note that the E-values are computed using the equation
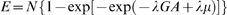
where 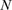 is the database size, 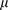 and 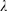 are the extreme value distribution (EVD) parameters of the domain model. The bottom plot depicts the histogram of the 10340 domains in Pfam rel.23. The median of all log E-values that corresponded to the domain-specific GAs is found to be −1.16. This translates to an E-value of 0.07.

Whereas the E-values in the order of 0.1 are generally considered being below the significance threshold (and they are for many good domain models as we observed in our practice), we find actually no general relationship between domain-specific gathering scores and E-value thresholds for Pfam release 23 (Figure 6). In fact, the gathering score thresholds can result in vastly different E-value thresholds (range 10^−35^ to 10^5^). Nevertheless, E-value thresholds close to the empirical value of 0.1 are most frequent in Pfam (see bottom part of Figure 6 with the peak of the E-value threshold histogram at 0.07) and one wonders why there are domains at all where the E-value corresponding to the gathering score does dramatically differ from 0.1. There might be many reasons for this discrepancy and its resolution would require dedicated research. It would be of interest to see how the growth of sequence databases as well as of the biological knowledge (in contrast to the more static seed alignments and domain models) has an effect here. We also suggest that, among other factors, incompleteness of the seed alignments with regard to the actual sequence variety (due to sequences that became available after model construction), alignment length (actually involving several domains in one model instead of one), the presence of non-globular segments or other issues of alignment quality might play a role here.

Lastly, E-values are comparable since they are a statistical measure but gathering score thresholds are not and, therefore, scores calculated from different domain models or prediction tools cannot be compared. This makes decisions among domain models and other prediction tools hitting the same segment in the query difficult. For example, the sequence XP_001939830.1 (Table S1, entry 19) illustrates this point. It is a hit in the fragment mode both by MFS_1 (over positions 49 to 388, E = 1.9e-21, score = 79.3>gatheringscore = 25.4) and HCV_NS4b (over overlapping positions 178 to 211, E = 4.8e-5, score = 14.6>gathering score = 14.5). Whereas the first is a full domain hit, the second one covers essentially only a TM region. Although both are above gathering score, the E-value clearly supports finding the correct annotation.

We do not want to create the impression that we wish to nail down the Pfam team on, maybe, some unfortunately selected thresholds for previous releases. Also, the specific examples (rather the existence of such examples) are not relevant for the conclusions in this paper. We have to live with some error rate. In contrast, it is important that the theoretical fundamentals are reliable, that systematic causes for possibly questionable annotations are increasingly suppressed and that, together with the Pfam team, the community develops the theory.

### About the state of automated annotation transfer in public databases

It is difficult to assess the total amount of wrong annotations currently persisting in public sequence databases since most of the protein sequences have never been a target of experimental study. With regard to theoretically derived function descriptions, the individual teams contributing to sequence databases, apparently, apply criteria with differing stringency and rigor. It appears that unrestrained annotation transfer justified by spurious sequence similarities is a major cause for annotation errors [64], [65] and this process is facilitated by the convenience of automated annotation pipelines. Analogous to a self-replicating virus, any first annotation error perpetually propagates itself to any existing or new sequence database by the virtue of annotation transfer ironically [64], [65].

In their analysis of database annotations for 37 enzyme families, Schnoes *et al.* [74] find approximately 40% of submitted sequences in 2005 were misannotated while none carried wrong annotation in 1993. It should emphasized that, in most cases, the misannotation involves an enzyme family or superfamily mix-up. To note, the fold as well as the overall function have been recognized correctly. We want to caution that the disregard of non-globular segments in context of homology-based conclusions can contribute to annotation errors. This may mean not just missing the correct subfamily but leading function assignment far astray. In the examples provided in this article, the true function of the protein hits has nothing in common with the problematic domain model hit except for the occurrence of a hydrophobic region that matches the SP/TM segment(s).

Thus, the criteria for sequence homology in their present form appear not directly applicable to non-globular segments. SPs/TMs as part of domain models lead to pollution of database annotations as our PIR iProClass v3.74 analysis demonstrates. As a matter of fact, it is very difficult to prove wrong annotation for experimentally uncharacterized sequences otherwise than by detecting logical contradictions. Whereas the examples in Tables S1 and S2 have been carefully scrutinized manually against structural and literature information, the same approach is out of question for a database-scale study, even for selected domain models as in Table 2. Therefore, we applied a criterion based on score partition into the SP/TM-specific part and the remainder to estimate the amount of false-positive hits to get at least a lower boundary estimate for the scale of the problem. We did show the existence of problematic annotations from a few to over ten percent for a validated set of 11 Pfam domains that include SP/TM regions.

### Conclusions

To conclude, sequence similarity among non-globular protein segments does not necessarily imply homology. Since matching of SPs/TMs creates the illusion of alignable hydrophobic cores, the inclusion of SPs/TMs into domain models without precautions can give rise to wrong annotations. We find that clearly more than 1001 domains among the 10340 models of Pfam release 23 suffer from this problem, whereas the issue is of relatively low importance for domains of SMART version 6 (18 out of 809). As expected, fragment-mode HMM searches generate promiscuous hits limited to solely the SP/TM part among clearly unrelated proteins for these models. More worryingly, we show explicit examples that the scores of clearly false-positive hits even in global-mode searches can be elevated into the significance range just by matching the hydrophobic runs. In the PIR iProClass database v3.74, we find that between 2.1% and 13.6% of its annotated Pfam hits appear unjustified for a set of validated domain models. We suggest a workflow of flagging problematic hits arising from SPs/TMs-containing models for critical reconsideration by annotation users. On the other hand, we have also seen that the inclusion of SP/TM regions into domain models can give rise to false negatives by imposing the need to have good scores over these regions in the query sequences when the actual domain occurs without the SP/TM context.

## Materials and Methods

### Assessment of false-positive detection of SP/TM segments by unsupervised prediction

It is well known that the problem of transmembrane helix prediction is not so much the detection of true hits as the suppression of false-positives [85]. In our context, it is important to have as few as possible wrong SP/TM predictions (and to carefully control their fraction) even on the expense of loosing true examples. Further, SP/TM prediction tools are designed for application to a single sequence, not to an alignment possibly polluted with gaps and/or shifts among predicted SP/TM regions among various sequences. Therefore, we developed the following procedure and statistical criteria for processing outputs of academically available SP/TM predictors.

In the general case, domain models are characterized by both seed and full alignments. We think that, in our context, operating with seed alignments is preferable since they are manually validated and are supposed to have lower levels of inclusions of unrelated sequences.

For a given domain model alignment, each sequence was subjected to sets of transmembrane (TM) and signal peptide (SP) segment predictors. We have used the following TM predictor tools – DASTM [85], [86], TMHMM [81], HMMTOP [87], SAPS [88], PhobiusTM [89], [90] and SP predictors – SignalP [29], [33], [91], PhobiusSig [89], [90]. The variable 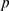 denotes the number of predictors in each set (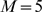 and 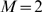 for TM and SP predictions respectively).

For each predictor 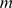, only the positive or negative SP/TM predictions for each residue 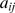 (where 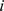 is the sequence and 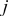 the alignment position) were considered, their respective prediction scores were ignored. Essentially, each positive/negative prediction can be seen as a Bernoulli random variable 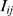 (an indicator variable assuming values one or zero). Collectively, a set of Bernoulli variables for each column 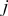 (made up by a number of sequences in the alignment) can be treated as a binomial random variable 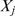 having the value 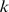 (sum of 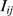 over all sequences 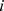).

To ensure that columns of domain alignments with an unequal number of sequences and/or gap instances are treated comparably, a hypothesis testing step is introduced [92]. Let 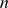 be the number of sequences (excluding gaps in the particular column) in the alignment. With 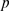, we denote the actual (*a priori* unknown) probability of the residue 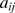 to belong to a true SP/TM segment. For each test, one wishes to determine if each column is a SP/TM residue given the observed predictions under equal chance condition. Hence, the null and the alternative hypotheses are stated as 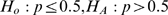. The type I error is defined as
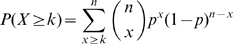
(1)


We assume the null hypothesis is rejected at a significance level of 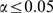. This means that, for alignments of four sequences and less, 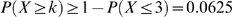 and, therefore, the null hypothesis is never rejected. The statistical test requires alignments of 5 sequences or more. For each rejected hypothesis, the corresponding expected positive predictions 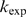 is calculated as
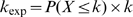
(2)Otherwise, 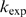 is set to zero. Finally, the estimated probability 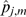 of column 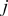 to represent a residue of a true SP/TM segment is given as
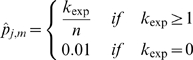
(3)The lower line in equation 3 is to avoid logarithms of zero in formulas below. Collectively, each domain alignment leads to a matrix of 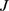 column probabilities 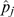 with 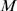 predictors for each segment type (TM or SP). The total logarithmic probability per column for either type of predictors is given as
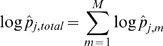
(4)If 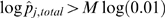, we assume that position 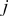 belongs to a predicted SP/TM segment. We define the indicator functions 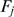 being unity in this case and zero in the other. Thus, a section of continuous alignment positions of unities in 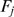 is called a predicted TM (or SP) segment. The average logarithmic probability 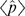 of this segment is given as
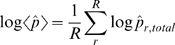
(5)where 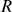 is the total number of predicted residue columns for the given SP/TM segment and 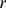 is the starting position of this TM helix or signal peptide.

In practice, some of the predicted transmembrane helices and signal peptides can be fragmented due to small gaps in the alignment. In the case of signal peptide fragments, it is reasonable to assume that all the fragments come from a single signal peptide. Consequently, the average logarithm probability of SP prediction per domain is simply calculated using (5) summing over the smallest region that contains both the N-terminal alignment position and the C-terminal boundary of the most C-terminal predicted segment.

However, for the case of the fragmented TM helices, the situation can be complicated by occurrences of multiple transmembrane segments within the alignment. As indicator which fragments to unite into one segment, we use the raw TM predictions. The indicator function 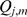 is set to unity at position 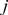 where predictor 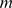 generates 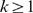 (union of the column-wise TM predictions in all sequences); otherwise, it is equal to zero. The composite indicator function 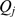 is set to unity only at positions 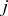 where 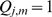 for all predictors that produce overlapping hits (intersection of predicted TM segments among all predictors). Similarly to predicted segments in 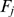, continuous runs of ones can be delineated in 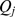. If two predicted segments in 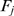 overlap with the same predicted segment in 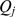, the zero values of 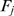 in-between the two segments are restored to unity. The union operation preserves the continuity within a helix while the intersection operation maintains separation between helices. Finally, the average logarithm probability 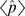 for a predicted TM segment consisting of 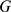 united fragments is given as weighted average
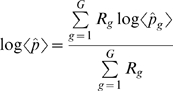
(6)where 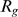 is the total number of predicted TM residue columns in the 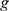 ^th^ TM helix fragment. Only predicted segments with a 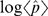 above a cutoff (*TMcutoff* or *SPcutoff* respectively; see below) are considered in the further analysis; others are discarded and the respective positions in 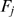 are set to zero.

We have used our algorithm also to find SP/TM regions in α- and membrane proteins classified by SCOP [41], [42] as a benchmark for finding TMcutoff. In this case, a single sequence and not an alignment is available; thus, we start with equation 3 and the conditions 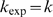 and 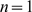.

### Specific considerations for transmembrane and signal peptide predictions

For the TM prediction problem, only the individual TM helix has been defined so far. To define a TM region that composes of one or more TM helices, adjacent TM helices separated by less than 40 amino acid residues are concatenated to form a region. The choice of 40 amino acids is based on the current knowledge that the smallest known globular domains such as Zinc fingers [93]–[97] are above 40 residues in length; thus, the inter-TM-helix residues just form some type of linker.

For the SP prediction problem, it is relevant that the actual N-terminus might be missing in the domain alignment. Thus, two rounds of SP predictions are necessary. After the initial round, the domain sequences with positive SP predictions are subjected to blastp runs (with parameters ‘-M BLOSUM62 -G 11 -E 1 -F F -I T’) against NR database to retrieve their full sequence data. Only the full sequence data with percent identity ≥95% and Blast E-value ≤0.01 are then subjected to SP predictions. Finally, only overlapped SP predictions that are confirmed in both rounds are retained for further processing.

### Determination of domain error cutoffs

The appropriate cutoff for predicted TM and SP segments in domain alignments have been determined with the help of the SCOP v1.75 [41], [42] α protein, membrane class database and SMART version 6 database [29], [40].

**Figure 7:**
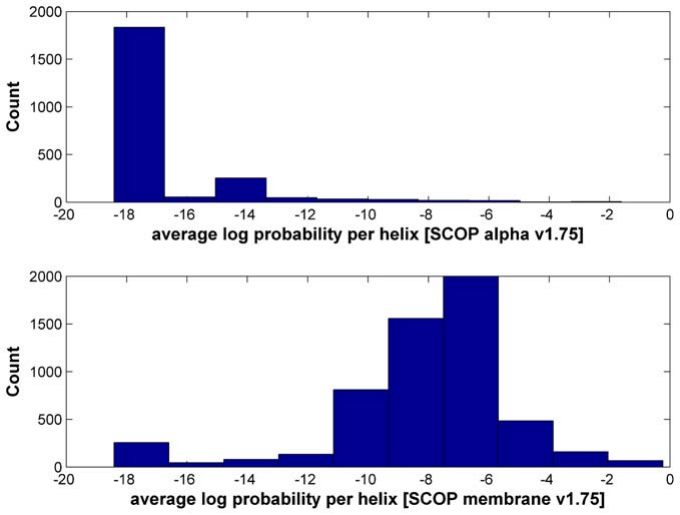
Histograms of average log probability per predicted transmembrane helix for SCOP v1.75 α-proteins class and membrane protein class. The top (average log probability per predicted transmembrane helix for SCOP v1.75 α-proteins class) and bottom (average log probability per predicted transmembrane helix for SCOP v1.75 membrane protein class) histograms represent the false-positive and true-positive distributions for TM predictions respectively. The total number of predicted structural and membrane helices is 2293 and 5592 respectively.

TM prediction hits among SCOP α class proteins are false-positives since the database contains predominantly structural helices. On the other hand, the membrane class contains mostly TM helices that made up the true-positive hits for these predictors. Figure 7 shows the histograms of the structural (top) and transmembrane (bottom) helices respectively. The clear separability between the two histograms strongly demonstrated that these two classes of helices are distinct. Table 3 gives the associated false-positive and false-negative rates of TM predictions at the various TM cutoffs.

**Table 3:** FP and FN rates of TM predictions based on different TM cutoffs.

Average log probability of TM prediction	No. of FP	FP rate (%)	No. of FN	FN rate (%)
≥−6	21	0.91	4519	80.81
≥−7	37	1.61	3401	60.82
≥−8	45	1.96	2520	45.06
≥−9	47	2.04	1593	28.49
≥−10	72	3.14	910	16.27
≥−11	84	3.66	526	9.41
≥−12	107	4.67	418	7.48
≥−13	125	5.45	381	6.81
≥−14	206	8.98	362	6.47

The first column gives the various cutoffs for the average log probability of TM helix prediction (refer to equations 5 and 6). The next two columns denote the number and percentage of false-positive TM helices with respect to 2293 predicted helices from SCOP α-proteins based on the corresponding cutoff rate. Similarly, the last two columns describe the number and percentage of false-negative TM helices with respect to 5592 predicted helices from SCOP membrane proteins.

**Figure 8:**
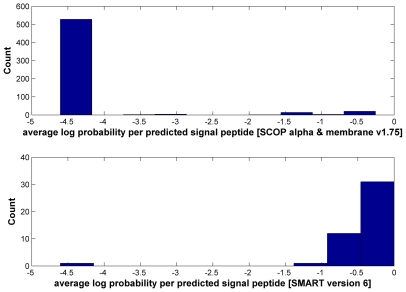
Histograms of average log probability per predicted signal peptide for SCOP v1.75 α- and membrane protein class and SMART version 6. The top (average log probability per predicted signal peptide for SCOP v1.75 α- and membrane protein class) and bottom (average log probability per predicted signal peptide for SMART version) histograms represent the false-positive and true-positive distributions for the SP predictions respectively. The total number of predicted signal peptides for SCOP α- and membrane proteins is 193 and 379 respectively, while the total number for SMART is 45. All except SM00817 Amelin (no available structure) were validated against their respective PDB entries.

In the case of the signal peptide prediction, both α- and membrane SCOP classes will deliver false-positive hits while the domain models from SMART with signal peptide are true positive hits. Figure 8 shows the histograms of false (top) and true signal peptides (bottom) respectively. In all, 45 out of 49 seed sequences for 5 SMART domains (SM00190 IL4_13, SM00476 DNaseIc, SM00770 ZN_dep_PLPC, SM00792 Agouti, SM00817 Amelin) were found to contain a predicted signal peptide. Out of them, predicted signal peptides for sequences from 4 domain models (except SM00817) were validated by their absence as a structural helix in the respective PDB entries (see Results, Table 1). Table 4 gives the associated false-positive and false-negative rates of SP predictions at different SP cutoffs.

**Table 4:** FP and FN rates of SP predictions based on different SP cutoffs.

Average log probability of SP prediction	No. of FP	FP rate (%)	No. of FN	FN rate (%)
≥−0.5	20	3.50	8	17.78
≥−1	23	4.02	1	2.2
≥−2	38	6.64	1	2.2
≥−3	38	6.64	1	2.2
≥−4	44	7.69	1	2.2

The first column gives the various cutoffs for the average log probability of SP prediction (refer to equation 5). The next two columns denote the number and percentage of false-positive SP with respect to 572 predicted SP from SCOP α- and membrane proteins based on the corresponding cutoff rate. Similarly, the last two columns describe the number and percentage of false-negative SP with respect to 45 predicted SP in seed sequences from SMART version 6 alignments.

### Decomposition of HMM log odd scores into sequence segment specific components

In the following, the reader is assumed to be familiar with chapter three of [47] and our derivations starts with a reformulated version of their equation 3.6. Let the observed and hidden state sequences be 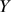 and 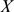. The joint probability of the observed and hidden state sequences is given as 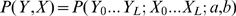 where 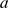 and 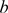 are the emission and state transition probabilities of the model, and 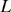 is the length of the sequence. Upon expanding the equation, we get
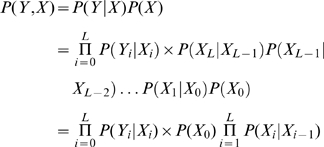
(7)The marginal probability of the observed sequence 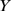 can be then be summed across all hidden sequence 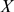 as
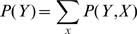
(8)Often the most probable path given by 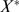 (given by the Viterbi algorithm) is a good approximation to 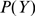. Hence we have
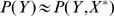
(9)In the HMM formalism, we use the log odd scores 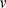 for scoring sequences. Therefore, for an observed sequence 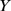, this is given as
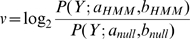
(10)Assume that 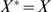. Using (7) to (10), the log odd score 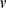 can be rewritten as
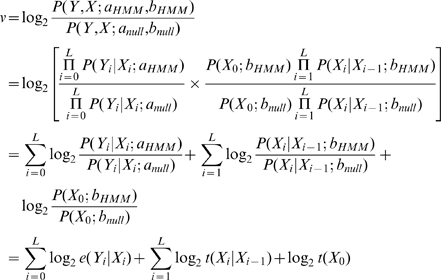
(11)where 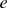 and 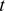 are the emission and state transition log odd scores. Thus, the total score is represented as a linear combination of sequence position-specific terms (plus some position-independent constants and, what is not considered here, the so-called null2 correction). Therefore, the HMM log odd score can be decomposed into sequence segment-specific contributions, for example those arising from its globular and non-globular regions:
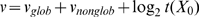
(12)where 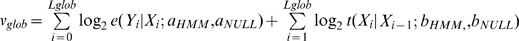,
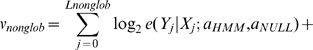


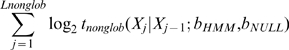
 are the total lengths of the globular and non-globular segments respectively; 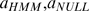 are the emission probabilities of the HMM and the null model respectively; 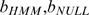 are the transition probabilities of the HMM and the null model respectively. In our work, we consider the SP/TM segments defined by 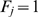 as non-globular part and the rest as globular.

### Estimation of the non-SP/TM component of the gathering score threshold

Here, equation (12) that denotes the total score 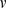 can be re-written as the sum of a non-SP/TM-specific 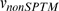, a SP/TM-specific 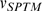, and a position-independent score 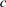 for a sequence as follows
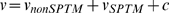
(13)In the following, we wish to derive the relative contribution of 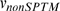 and 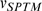 at scores 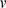 close to the gathering score 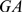. We assume that the proportion between 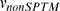 and 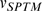 as represented by the sequences from the seed alignment holds also for lower scores of true hits. Let the random variables 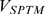 and 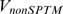 denote the SP/TM-specific scores 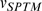 and non-SP/TM specific scores 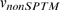 of 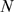 seed sequences of the domain model. The sample mean 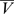 of the random variables are given as
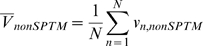
(14)

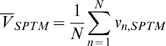
(15)Here, we introduce a scaling factor in the form of random variable 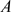 as a shift factor in the logarithmic scale that relates the random variables 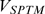, 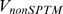 and the constant 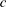 to the constant 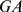 (gathering score threshold provided the domain model). The relationship can be written as
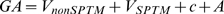
(16)Equation (16) can further be expressed in terms of two random variables 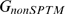 and 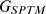 that denote the SP/TM-specific and non-SP/TM-specific gathering score threshold means respectively.
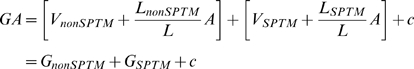
(17)To obtain the mean of 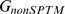, we first need to solve for 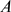 by rewriting equation (16) in terms of 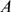 as given
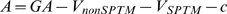
(18)Consequently, taking the expectation of 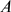 (the sample mean over the seed alignment), we get
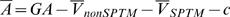
(19)Finally, the non-SP/TM specific contribution 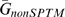 to the gathering score threshold is given as
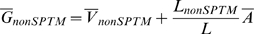
(20)Similarly, a SP/TM-specific threshold 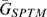 can be calculated. For the 11 domain models in Table 2, 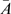 is vastly negative and ranges from −76.19 (Cation_ATPase_N) to −614.95 (Lamp); thus, 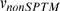 is much larger than 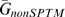 for any seed sequence.

### Estimation of unjustified annotation instances in the database

For a set of sequences with a common problematic domain annotation, each sequence score can be represented by 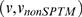. If we assume that all true hits must score above the gathering score 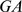 and the threshold 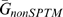 as derived in the previous section is truly the lower boundary for a score contribution from the non-SP/TM part of a correct domain hit, the validity of the annotation can be assessed by comparing 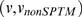 with 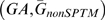. If 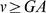 and 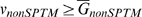, the domain hit is considered true-positive. If 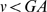 and 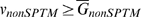, the SP/TM part of the domain hit is degenerated; yet, the non-SP/TM part is well represented and we consider these hits false-negatives. In all cases with 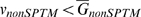, we consider the annotation with the domain unjustified. Even if the total score is above the gathering score, formally, the shift to the significant range is only achieved by a large score from the SP/TM region.

We find that our derivation for 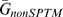 is credible since it does not compromise the sensitivity of the domain models. The fraction of false-negative hits over the total retrieved sequences per problematic domain ranges between 0 to 5% (with the only outlier GRP at 10.1%).

## Supporting Information

Figure S1 PF00583 hits leading to the Eco1 function discovery.
(0.03 MB PDF)

Figure S2 False-positive hit of PF00497 in Alt a 1.
(0.01 MB PDF)

Protocol S1 Mini-site with supplementary information, archive created with WinRAR (to be downloaded from http://www.rarlab.com/download.htm). Besides HMMER outputs, alignments, etc. for Tables S1, S2 and 3 and for Figures 4 and 5, we provide lists of affected Pfam models as well as HMMs for these domains without the respective SP/TM segments. The content of this file may also be found at http://mendel.bii.a-star.edu.sg/SEQUENCES/ProblemDomains-TMSP/.
(32.83 MB WinRAR)

Table S1 Summary of selected sequence hits with problematic domain annotations (fragment-mode search).
(0.04 MB PDF)

Table S2 Summary of selected sequence hits with problematic domain annotations (global-mode search).
(0.05 MB PDF)

Table S3 Summary of selected false-negative sequence hits with problematic domain annotations (global-mode search).
(0.05 MB PDF)
