# Supplementary material for: More Than 1,001 Problems with Protein Domain Databases: Transmembrane Regions, Signal Peptides and the Issue of Sequence Homology
Source: PLoS Comput Biol. 2010 Jul 29;6(7):e1000867. doi: 10.1371/journal.pcbi.1000867 (PMC2912341; doi:10.1371/journal.pcbi.1000867)
Supplement: Figure S1 — PF00583 hits leading to the Eco1 function discovery. (0.03 MB PDF) [file pcbi.1000867.s001.pdf]

Supplementary Figure S1
Fragment-mode HMMER2 hits of the Acetyltransf domain model into Eco1 homologous sequences

```
>ECO1A_ORYZA_SATIVA GI|5702976|GB|AQ794507.1|AQ794507.[91:444].SP.TR GENEWISE
TRANSLATIONVYLFISSQRIVGCLVVEPIKTAHKVIPGSTEENGKYRDPGAIICEKEDVPAHCGFRAIWV
VPSRRRKRIGSQLMDAAR


HMMER 2.1.1 (Dec 1998)

- - - - - - - - - - - - - - - - - - - - - - - - - - - - - - - - - - - - -

Query:  ECO1A_ORYZA_SATIVA  GI|5702976|GB|AQ794507.1|AQ794507.[91:444].SP.TR GENEWISE TRANSLATION



Acetyltransf   Acetyltransferase (GNAT) family           13.5     0.0017   1

Acetyltransf     1/1       2    77 ..     1    46 [.    13.5   0.0017



Acetyltransf: domain 1 of 1, from 2 to 77: score 13.5, E = 0.0017

                   *->lvaeedgelvGfaglspide..........................

                      ++     ++vG+  + pi   ++  +++++++++ ++++    ++++

  ECO1A_ORYZ     2    YLFISSQRIVGCLVVEPIKTahkvipgsteengkyrdpgaiiceked 48



                   ...vaeieglaVdpeyRgkGiGtaLleal<-*

                    + ++   +++V p+ R+k iG++L++a+

  ECO1A_ORYZ    49 vpaHCGFRAIWVVPSRRRKRIGSQLMDAA    77

>ECO1_CA ORF6.3383.PROT  ORF6-2238:8014-8796:E  783 BP, 260 AA, CONTIG 17099 BP [CANDIDA ALBICANS]
MGSINSQKPQKIQSILALPSNFKKITCSTCDMTYNPHISQDKLLHNKYHTNFINGIPWNYKTDNDVLIIENFTLVETPKLNSTGKSLKSTKTRQTFKGSI
ICINKSNKRHIQKVELLLNMVNQELNASQDSGQWKKPEFDRSKAFVIIIDSKAIGLCTTDTIQPDQGRWMIHKTQSIVPNQINKNVVIGISRIWISRKWR
QYGLGKKLLNVVLKNSIYSVQLLKNQVAFSQPSFSGGMLAKSFNGVKHKSGEMLLPVYIE


HMMER 2.1.1 (Dec 1998)
- - - - - - - - - - - - - - - - - - - - - - - - - - - - - - - - - - - - -
Query:  ECO1_CA  ORF6.3383.PROT  ORF6-2238:8014-8796:E  783 BP, 260 AA, CONTIG 17099 BP [CANDIDA
ALBICANS]

Acetyltransf Acetyltransferase (GNAT) family             11.4      0.009   1
Acetyltransf   1/1     145   214 ..     1    48 [.    11.4    0.009

     ECO1_CA    37    HISQDKLLHNKYHTNFINGI    56

Acetyltransf: domain 1 of 1, from 145 to 214: score 11.4, E = 0.009
                   *->lvaeedgelvGfaglspide.....................vaeie
                      +v+ +d + +G ++   i++++++   +++++  +++ +++ v+ i
     ECO1_CA   145    FVIIIDSKAIGLCTTDTIQPdqgrwmihktqsivpnqinknvVIGIS 191

                   glaVdpeyRgkGiGtaLlealee<-*
                   ++++++ +R  G+G++Ll+ +++
     ECO1_CA   192 RIWISRKWRQYGLGKKLLNVVLK    214

>ECO1_CIONA_INTESTINALIS GI_1 NORI SATOH UNPUBLISHED CDNA LIBRARY CIONA INTESTINALIS CDNA CLONE
RCITB3J17 3'

HDQHQHKLHHQRFNVILRFPSWKNERTVASYVDGRIVKILPTDPKFAQKKVEDILELIDSELGFSQNISTTRVSYLYVSDQQQVTGCLIAEQIKRGFPLL
ETMTSSGMMSCSLQSTPVTCGVSRIWCHAPHRKRGVATRLMDALRCSFVLGERLNMNQVAFSDPTISGKSFATKYFKTPH
FLTYNCST


HMMER 2.1.1 (Dec 1998)

- - - - - - - - - - - - - - - - - - - - - - - - - - - - - - - - - - - - -
```

Query: ECO1_CIONA_INTESTINALIS GI_1 NORI SATOH UNPUBLISHED CDNA LIBRARY CIONA INTESTINALIS CDNA CLONE RCITB3J17 3'

```
Acetyltransf Acetyltransferase (GNAT) family             11.9     0.0064   1

Acetyltransf   1/1     107   144 ..    11    46 ..    11.9   0.0064



Acetyltransf: domain 1 of 1, from 107 to 144: score 11.9, E = 0.0064

                   *->Gfaglspide..vaeieglaVdpeyRgkGiGtaLleal<-*

                      G++++s    + ++ + +++ +  +R++G++t+L++al

  ECO1_CIONA   107    GMMSCSLQSTpvTCGVSRIWCHAPHRKRGVATRLMDAL    144
```

>ECO1_DM GI|7295257|GB|AAF50579.1| (AE003559) CG8598 GENE PRODUCT [DROSOPHILA MELANOGASTER]
METPTGSGRPSRMATPRLSERKRQLFGSPRSRLRQINDDEDDADVDSLGVLPLKTHVAANRKGRSLFAAVPGKSSSSANSSPETNKENKKTRGGVMTATA
EQLPQLFTATMRLNSNSSSNSRNSSPRQTRVQRKRADSSMSSPTSSSEGTPSSRARNSIRRSPRTFSAQKDPDAFSSPESFQTRLSKVAAMLMKGQDSRS
MLEKSKKKHNHSLKTTAQVHTTKPKKTSPAEESQSDDEKPSSSKNSRKNTEVRETRSSQIISPKTRNRRRPFTSADINCKTLKAAAHLHENMRSYDEEKT
AAVKLENSRSRSKSPVEVFKSNDDAVKRNTGNTNNKTAKSSEVATAKRPESPGSSMKIDVEVPESDEEASNHKPQKRQHPETSTPVAPSADADSGSPQSK
MRKVTLSSSIPTMAFYSHSGEAVTKSRRRPSISKNSLKQPTKISPTSRPLLGINKGVHHKIRKRHGFANRLPATDMDNILNSLSNERLKNLITTKREERA
KVEEVHQILRNAKDPIKMAKPLSVIEADDANNNNNLPATAWQETSADFSDLSDVEDIDPIIEVEPIIPIIRHEPVQKSPTAEPADLSKRKFFKSGRRSST
CMEVRITDNIRASVSQGKIELVQTIRRKPRQVRVKSATIFSAEQATVDAILKNLDDTVVDEIVEANPVVQATPIDAEETTMETESLPDIIEYAPEANDVE
IDPFAEFRQRLPYQTDDPNVVEQQQILLEFLISNNICTEKNFEIFIANPDDYKDEANQIVDNLYMVVNSEEAAQLAQMETVENTAVAIAPKQDAPAVEEV
QPKLFPIFTQRLQPVVQKSLRRRPDTSMRLLTAAGGSNQYQIDAGQKAFGARQCQQCGLVYTVHEPEEELLHREYHNSIHVLRFKGWIDEDIVSVYPEWA
SDGRIIRINERAPTARLDRLRDLIGVVDKELGYSSYIVPKIFVAFIAVRKQQIVGFCLVQPLSQAHRFIQVDGTDYFSEESYPASCGVSRIWVSPLQRRS
GIASKLLRVVQCHTVLGQEIARECIAFSTPTDDGRALARQFTGLDNFLTYDQ

HMMER 2.1.1 (Dec 1998)

- -

Query: ECO1_DM GI|7295257|GB|AAF50579.1| (AE003559) CG8598 GENE PRODUCT [DROSOPHILA MELANOGASTER]

```
Acetyltransf  Acetyltransferase (GNAT) family            17.5    6.4e-05   1
Acetyltransf    1/1     945  1008 ..     1    44 [.    17.5  6.4e-05


Acetyltransf: domain 1 of 1, from 945 to 1008: score 17.5, E = 6.4e-05

                   *->lvaeedgelvGfaglspide...................vaeiegl

                      ++a ++ ++vGf+ + p  + ++  + ++++  ++++ +  + + ++

     ECO1_DM   945    FIAVRKQQIVGFCLVQPLSQahrfiqvdgtdyfseesypaSCGVSRI 991



                   aVdpeyRgkGiGtaLle<-*

                   +V+p  R+ Gi+++Ll+

     ECO1_DM   992 WVSPLQRRSGIASKLLR    1008
```

>ECO1_SC GI|6321140|REF|NP_011218.1| INVOLVED IN ESTABLISHMENT OF COHESION BETWEEN SISTER CHROMATIDS; ECO1P#GI|1176006|SP|P43605|YFJ7_YEAST HYPOTHETICAL 31.8 KD PROTEIN IN HIS2-CDC14 INTERGENIC REGION#GI|1084747|PIR||S56282 HYPOTHETICAL PROTEIN YFR027W - YEAST (SACCHAROMYCES CEREVISIAE)#GI|836782|DBJ|BAA09266.1| (D50617) YFR027W [SACCHAROMYCES CEREVISIAE]#GI|893422|DBJ|BAA08038.1| (D44602) UNKNOWN [SACCHAROMYCES CEREVISIAE]

MKARKSQRKAGSKPNLIQSKLQVNNGSKSNKIVKCDKCEMSYSSTSIEDRAIHEKYHTLQLHGRKWSPNWGSIVYTERNHSRTVHLSRSTGTITPLNSSP
LKKSSPSITHQEEKIVYVRPDKSNGEVRAMTEIMTLVNNELNAPHDENVIWNSTTEEKGKAFVYIRNDRAVGIIIIENLYGGNGKTSSRGRWMVYDSRRL
VQNVYPDFKIGISRIWVCRTARKLGIATKLIDVARENIVYGEVIPRYQVAWSQPTDSGGKLASKYNGIMHKSGKLLLPVYI

HMMER 2.1.1 (Dec 1998)

- -

Query: ECO1_SC GI|6321140|REF|NP_011218.1| INVOLVED IN ESTABLISHMENT OF COHESION BETWEEN SISTER CHROMATIDS; ECO1P#GI|1176006|SP|P43605|YFJ7_YE

Acetyltransf Acetyltransferase (GNAT) family 13.9 0.0012 1

Acetyltransf 1/1 162 236 .. 1 48 [. 13.9 0.0012

Acetyltransf: domain 1 of 1, from 162 to 236: score 13.9, E = 0.0012

```
                   *->lvaeedgelvGfaglspide..........................
                      +v+ ++++ vG + +   ++++++++++++    ++++  ++  ++
     ECO1_SC   162    FVYIRNDRAVGIIIIENLYGgngktssrgrwmvydsrrlvqnvypdf 208


                   vaeieglaVdpeyRgkGiGtaLlealee<-*
                    + i +++V + +R+ Gi+t+L++ + e
     ECO1_SC   209 KIGISRIWVCRTARKLGIATKLIDVARE    236
```

>ECO1_SP GI|7490455|PIR||T39541 DNA DAMAGE INDUCIBLE PROTEIN HOMOLOG - FISSION YEAST (SCHIZOSACCHAROMYCES POMBE)#GI|2842510|EMB|CAA16862.1| (AL021748) SIMILAR TO DNA-DAMAGE-INDUCIBLE PROTEIN [SCHIZOSACCHAROMYCES POMBE]#GI|7678718|DBJ|BAA95122.1| (AB039861) ESO1 [SCHIZOSACCHAROMYCES POMBE]

MELGKSKFSWKDLQYCDKAGTQNSPLRVVAHIDQDAFYAQVESVRLGLDHSVPLAVQQWQGLIAVNYAARAANISRHETVTEAKKKCPELCTAHVKTWKA
GESEAKYHENPNPNYYKTCLDPYRHESVKILNIIKKHAPVVKKASIDECFIELTSDVKRIVLEEYPYLKIPSEDSNVALPQAPVLLWPAEFGMVIEEEVV
DRTKEDYERDWDDVFLFYAAKIVKEIRDDIYLQLKYTCSAGVSFNPMLSKLVSSRNKPNKQTILTKNAIQDYLVSLKITDIRMLGGKFGEEIINLLGTDS
IKDVWNMSMDFLIDKLGQTNGPLVWNLCHGIDNTEITTQVQIKSMLSAKNFSQQKVKSEEDAINWFQVFASDLRSRFLELEGMRRPKTICLTVVSRFLRK
SRSSQIPMNVDISTQFIVEATSKLLRQLQQEFDVYPISNLSISFQNIIEVDRNSRGIEGFLKKSNDEIYMSTSVSPSIEGRAKLLNENMRENNSFELSSE
KDIKSPKRLKRGKGKGIFDMLQQTAVSKPTENSADETYTCEECEQKITLSERNEHEDYHIALSISRKERYNNLVPPSHDKPKQVKPKTYGRKTGSKHYAP
LSDETNNKRAFLDAFLGNGGNLTPNWKKQTPKAISNSSDNMTQLHLDLANSTVTCSECSMEYNSTSEEDILLHSRFHSRVLGGVTVSFQCSPIYRVNYGL
SSDCIYSINSESSLIDQRKAEEALSFVNNELSSEPIETIGVDKYTTFLFISDKKCVGLLLAERISSAYIVDELELNNNNSTSSAVYIKNENLRKGFVLGI
SRIWVSASRRKQGIASLLLDNALKKFIYGYVISPAEVAFSQPSESGKQFIISWHRSRNNGSSKSRYAVYES

HMMER 2.1.1 (Dec 1998)

- -

Query: ECO1_SP GI|7490455|PIR||T39541 DNA DAMAGE INDUCIBLE PROTEIN HOMOLOG - FISSION YEAST (SCHIZOSACCHAROMYCES POMBE)#GI|2842510|EMB|CAA16862

Acetyltransf Acetyltransferase (GNAT) family 9.9 0.03 1
Acetyltransf 1/1 784 825 .. 13 49 .. 9.9 0.03

>ECO1_YARROWIA_LIPOLYTICA GI_1 T7 END OF CLONE AW0AA013E09 OF LIBRARY AW0AA FROM STRAIN CLIB 89 OF YARROWIA LIPOLYTICA, GENOMIC SURVEY SEQUENCE

MKTYRAKRKYLSESEDDVFSSSPTQSPETSPLQPPNESRLNIKAAQAVSPCQKRAKVVKKPAKTKAPVQMTLSLGQTTSTTCKTCGMTYQVAYGPDISAH
KSFHSTALNGPKWKPSVSAVVVDKSKTYTVYKSRLLSHPCVSQFLKLVNSELNAPEPILSSQAAVYVYVVDQRAVGCVLVDRITKCRHVDIQTGTLGLKE
YPAVMGVSRMYVSQLFRRTGIVTKLLDLAKSDFIYGIGA

HMMER 2.1.1 (Dec 1998)

- -

Query: ECO1_YARROWIA_LIPOLYTICA GI_1 T7 END OF CLONE AW0AA013E09 OF LIBRARY AW0AA FROM STRAIN CLIB 89 OF YARROWIA LIPOLYTICA, GENOMIC SURVEY SEQUENCE

Acetyltransf Acetyltransferase (GNAT) family 12.7 0.0032 1

Acetyltransf 1/1 166 229 .. 1 46 [. 12.7 0.0032

Acetyltransf: domain 1 of 1, from 166 to 229: score 12.7, E = 0.0032

```
                   *->lvaeedgelvGfaglspide.................vaeieglaV
                      +v+ +d + vG++ +  i   ++ + ++++ + ++ + v  + +++V
```

```
ECO1_YARRO   166    YVYVVDQRAVGCVLVDRITKcrhvdiqtgtlglkeypaVMGVSRMYV 212

                 dpeyRgkGiGtaLleal<-*
                 +  +R+ Gi t+Ll+ +
ECO1_YARRO   213 SQLFRRTGIVTKLLDLA    229
```

Supplementary Figure 1B
Fragment-mode HMMER2 hits of the SBP_bac_3 (PF00497) domain model into the “Alt a I” sequence

```
>gi|1773369|gb|AAB40400.1| Alt a I subunit [Alternaria alternata]

MQFTTIASLFAAAGLAAAAPLESRQDTASCPVTTEGDYVWKISEFYGRKPEGTYYNSLGFNIKATNGGTLDFTCSAQADKLEDHKWYSCGENSFMDFSFD
SDRSGLLLKQKVSDDITYVATATLPNYCRAGGNGPKDFVC
QGVADAYITLVTLPKSS

HMMER 2.1.1 (Dec 1998)

Query:  gi|1773369|gb|AAB40400.1|  Alt a I subunit [Alternaria alternata]


SBP_bac_3     Bacterial extracellular solute-binding      7.0      0.072   1

SBP_bac_3       1/1       1    38 [.     1    60 [.     7.0    0.072


SBP_bac_3: domain 1 of 1, from 1 to 38: score 7.0, E = 0.072

                   *->MKklllagiWillllalallaalalagsgaaaaaldkeddtdlwekIk

                      M+++++a     +l  +++a la+a+++++ +++

  gi|1773369     1    MQFTTIA-----SL--FAAAGLAAAAPLESRQDT------------- 27


                   kkgvlrvaTegtY<-*

                     ++  v+Teg+Y

  gi|1773369    28 --ASCPVTTEGDY    38
```
