# Supplementary material for: More Than 1,001 Problems with Protein Domain Databases: Transmembrane Regions, Signal Peptides and the Issue of Sequence Homology
Source: PLoS Comput Biol. 2010 Jul 29;6(7):e1000867. doi: 10.1371/journal.pcbi.1000867 (PMC2912341; doi:10.1371/journal.pcbi.1000867)
Supplement: Figure S2 — False-positive hit of PF00497 in Alt a 1. (0.01 MB PDF) [file pcbi.1000867.s002.pdf]

Supplementary Figure S2
Fragment-mode HMMER2 hits of the SBP_bac_3 (PF00497) domain model into the “Alt a I” sequence

```
>gi|1773369|gb|AAB40400.1| Alt a I subunit [Alternaria alternata]

MQFTTIASLFAAAGLAAAAPLESRQDTASCPVTTEGDYVWKISEFYGRKPEGTYYNSLGFNIKATNGGTLDFTCSAQADKLEDHKWYSCGENSFMDFSFD
SDRSGLLLKQKVSDDITYVATATLPNYCRAGGNGPKDFVC
QGVADAYITLVTLPKSS

HMMER 2.1.1 (Dec 1998)

Query:  gi|1773369|gb|AAB40400.1|  Alt a I subunit [Alternaria alternata]


SBP_bac_3     Bacterial extracellular solute-binding      7.0      0.072   1

SBP_bac_3       1/1       1    38 [.     1    60 [.     7.0    0.072


SBP_bac_3: domain 1 of 1, from 1 to 38: score 7.0, E = 0.072

                   *->MKklllagiWilllalallaalalagsgaaaaaldkeddtdlwekIk

                      M+++++a     +l  +++a la+a+++++ +++

  gi|1773369     1    MQFTTIA-----SL--FAAAGLAAAAPLESRQDT------------- 27


                   kkgvlrvaTegtY<-*

                     ++  v+Teg+Y

  gi|1773369    28 --ASCPVTTEGDY    38
```
