# Supplementary material for: More Than 1,001 Problems with Protein Domain Databases: Transmembrane Regions, Signal Peptides and the Issue of Sequence Homology
Source: PLoS Comput Biol. 2010 Jul 29;6(7):e1000867. doi: 10.1371/journal.pcbi.1000867 (PMC2912341; doi:10.1371/journal.pcbi.1000867)
Supplement: Table S1 — Summary of selected sequence hits with problematic domain annotations (fragment-mode search). (0.04 MB PDF) [file pcbi.1000867.s004.pdf]

Supplementary file: Table S1. Summary of selected sequence hits with problematic domain annotations (Fragment-mode search)

Domain Name	Type, predicted region of alignment	Validated TM helices/ SP of model, reference	Sequence accession no. (No. of AA)	Sequence Description/ Taxonomy	Range of FP hits in sequence	Raw score/ E-value of FP hits with HMMER2
PF01537.9 : Herpes_glycop_D (Herpesvirus glycoprotein D)<br><br>Gathering score : 21<br>Alignment length: 410<br>HMM length: 409	TM,371-392	372-393<br><br>ref.[1]	**1. EAZ04186.1 (682 AA)**<br>*EEC82190.1(658AA)*	hypothetical protein OsI_025418, *Oryza sativa (indica cultivar-group)*	**252-324**<br>*279-300*	**27.2/3.5e-08**<br>*7.9/2.2e-2*
			**2. AAR96255.1 (364 AA)**	MHC class Ib antigen, *Rattus norvegicus*	**298-351**	**25.5/1.1e-07**
			**3. EEA49333.1 (1324 AA)**<br>*XP_002602522.1(1324AA)*	hypothetical protein BRAFLDRAFT_93828, *Branchiostoma floridae*	**620-664**	**24.7/2e-07**
			**4. XP_001519017.1 (849 AA)**	PREDICTED: similar to poliovirus receptor related 2, *Ornithorhynchus anatinus*	**720-776**	**19.6/6.5e-06**
			**5. XP_002124481.1 (693)**	PREDICTED: similar to Notch, *Ciona intestinalis*	**136-246**	**19.9/5.4e-06**
			**6. XP_001705150.1 (801 AA)**	High cysteine membrane protein Group 2, *Giardia lamblia ATCC 50803*	**755-795**	**18.3/1.7e-05**
			**7. XP_001349744.1 (369 AA)**	Rifin, *Plasmodium falciparum 3D7*	**311-364**	**17.3/3.3e-05**
			**8. XP_001619472.1 (610 AA)**	hypothetical protein NEMVEDRAFT_v1g224148, *Nematostella vectensis*	**423-463**	**15.7/1e-04**
PF03381.7 : CDC50: (ligand-effect modulator 3/CDC50 family)<br><br>Gathering score : 19.7<br>Alignment length: 495<br>HMM length: 353	TM,453-478	318-340<br><br>ref.[2]	**9. XP_001489157.1 (336 AA)**	PREDICTED: similar to carbonic anhydrase XIV, *Equus caballus*	**297-325**	**17.7/7.1e-05**
			**10. XP_540298.2 (337 AA)**	PREDICTED: similar to carbonic anhydrase XIV precursor, *Canis familiaris*	**298-326**	**13.7/9.8e-04**
			**11. XP_001924916.1 (233 AA)**	PREDICTED: similar to carbonic anhydrase 14, *Sus scrofa*	**85-175**	**14.5/5.8e-04**
			**12. ZP_02211465.1 (285 AA)**	hypothetical protein CLOBAR_01078, *Clostridium bartlettii DSM 16795*	**242-272**	**17.8/6.7e-05**
			**13. XP_001306144.1 (569 AA)**	polymorphic outer membrane protein, *Trichomonas vaginalis G3*	**479-511**	**14.4/6.5e-04**

PF00690.18 : Cation_ATPase_N (Cation transporter/ATPase, N-terminus)<br><br>Gathering score : 18.8<br>Alignment length: 107<br>HMM length: 87	TM, 85-105	TM,66-87<br><br>ref.[3]	**14. YP_001179318.1 (838 AA)**	1A family penicillin-binding protein, *Caldicellulosiruptor saccharolyticus DSM 8903*	**12-48**	**18.9/1.1e-4**
PF00482.11 : GSPII_F (Bacterial type II secretion system protein F domain)<br><br>Gathering score : 17.8<br>Alignment length: 185<br>HMM length: 136	TM, 166-184	TM,118-136<br><br>ref. [4]	**15. YP_253646.1 (442 AA)**<br><br>**16. YP_964038.1 (540 AA)**	hypothetical protein SH1731, *Staphylococcus haemolyticus JCSC1435*<br><br>methyl-accepting chemotaxis sensory transducer, *Shewanella sp. W3-18-1*	**157-196**<br><br>**157-206**	**18.0/4.4e-4**<br><br>**17.6/5.5e-4**
PF01569.13 : PAP2 (type 2 phosphatidic acid phosphatase)<br><br>Gathering score : 16<br>Alignment length: 261<br>HMM length: 177	TM,200-261	129-143, 156-172<br><br>ref.[5]	**17. AAP92612.1 (1640 AA)**<br><br>**18. XP_418136.2 (1153 AA)**	Ab2-371, *Rattus norvegicus*<br><br>PREDICTED: similar to Aoc2 protein, *Gallus gallus*	**1266-1323**<br><br>**945-1000**	**36.7/1.2e-09**<br><br>**35.6/2.5e-09**
PF01001.11 : HCV_NS4b (Hepatitis C virus non-structural protein NS4b)<br><br>Gathering score : 14.5<br>Alignment length: 199<br>HMM length: 199	TM,124-179	124-143, 156-179<br><br>ref.[6]	**19. XP_001939830.1 (440 AA)**	conserved hypothetical protein, *Pyrenophora tritici-repentis Pt-1C-BFP*	**178-211**	**14.6/4.8e-05**

In the first column, we list selected Pfam domains with their accession, identifier, description and their gathering score (as in Pfam release 23) that have TM regions included into the model. We also provide alignment length and the HMM length. The latter might be considerably shorter than the former as a result of hmmbuild defaults in HMMER2.

The region in the domain alignment that includes the predicted TM segments (together with interlinking loops as described in Methods) is provided in the second column. We searched for experimental proof of these predictions in the literature and the corresponding references and the positional ranges for the respective SP/TM segments (with respect to the HMM but not the alignment) are given in the third column.

The next two columns provide running number, accession (in bold), sequence length (in bold), description and taxonomic origin of sequences that were found as false-positive hits of the respective HMMs when using HMMER2 in the fragment-mode search. The penultimate column shows the range of the hit in the subject sequence (at the domain side, the hit was always over the full length of

the HMM, in bold font). The last column provides score and E-value for HMMER2 (in bold).

During the revision of this manuscript, several sequence entries have been updated. In these cases, the old sequence entries have been complemented by new accession numbers (in italic). Any subsequent changes affecting computational results (with regard to their corresponding positional changes, raw scores and E-values) are also provided in italic font if applicable.

Additional material such as hmmpfam outputs and alignments are available at the associated BII WWW site for this work.

References

1. Carfi A, Willis SH, Whitbeck JC, Krummenacher C, Cohen GH, Eisenberg RJ, Wiley DC (2001) Herpes simplex virus glycoprotein D bound to the human receptor HveA. Mol Cell 8: 169-179.

2. Misu K, Fujimura-Kamada K, Ueda T, Nakano A, Katoh H, Tanaka K (2003) Cdc50p, a conserved endosomal membrane protein, controls polarized growth in Saccharomyces cerevisiae. Mol Biol Cell 14: 730-747.

3. Xu C, Rice WJ, He W, Stokes DL (2002) A structural model for the catalytic cycle of Ca(2+)-ATPase. J Mol Biol 316: 201-211.

4. Abendroth J, Mitchell DD, Korotkov KV, Johnson TL, Kreger A, Sandkvist M, Hol WG (2009) The three-dimensional structure of the cytoplasmic domains of EpsF from the type 2 secretion system of Vibrio cholerae. J Struct Biol 166: 303-315.

5. Sun L, Gu S, Sun Y, Zheng D, Wu Q, Li X, Dai J, Dai J, Ji C, Xie Y, Mao Y (2005) Cloning and characterization of a novel human phosphatidic acid phosphatase type 2, PAP2d, with two different transcripts PAP2d_v1 and PAP2d_v2. Mol Cell Biochem 272: 91-96.

6. Qu L, McMullan LK, Rice CM (2001) Isolation and characterization of noncytopathic pestivirus mutants reveals a role for nonstructural protein NS4B in viral cytopathogenicity. J Virol 75: 10651-10662.
