# Supplementary material for: More Than 1,001 Problems with Protein Domain Databases: Transmembrane Regions, Signal Peptides and the Issue of Sequence Homology
Source: PLoS Comput Biol. 2010 Jul 29;6(7):e1000867. doi: 10.1371/journal.pcbi.1000867 (PMC2912341; doi:10.1371/journal.pcbi.1000867)
Supplement: Table S2 — Summary of selected sequence hits with problematic domain annotations (global-mode search). (0.05 MB PDF) [file pcbi.1000867.s005.pdf]

## Supplementary file: Table S2. Summary of selected sequence hits with problematic domain annotations (Global-mode search)

| Domain Name | Type, predicted region of alignment | Validated TM helices /SP of model, reference | Sequence accession no. (No. of AA) | Sequence Description/ Taxonomy | Range of FP hits in sequence | Raw score/ E-value of FP hits with HMMER2 (HMMER3) |
|---|---|---|---|---|---|---|
| PF08510.4 :<br>PIG-P<br>(phosphatidylinositol N-acetylGlucosaminyl transferase subunit P)<br><br>Gathering score : -11.4<br>Alignment length: 208<br>HMM length: 153 | TM,1-91 | 8-24,<br>44-67<br><br>ref.[1] | **1. EAY79580.1 (899 AA)**<br>EEC67477.1 (720AA)+*EEC67476.1 (163AA)*<br><br>**2. EAZ17037.1 (877 AA)**<br>EEE51441.1(720AA)+*EEC67476.1(163AA)*<br><br>**3. XP_001842924.1 (165 AA)**<br><br>**4. XP_761344.1 (379 AA)** | hypothetical protein OsI_033539, *Oryza sativa*<br><br>hypothetical protein OsJ_031246, *Oryza sativa*<br><br>conserved hypothetical protein, *Culex quinquefastiatus*<br><br>hypothetical protein UM05197.1, *Ustilago maydis 521* | **764-894**<br>*28-158*<br><br>**742-872**<br>*28-158*<br><br>**85-164**<br><br>**297-379** | **92.8/1.2e-24 (6.4e-23)**<br><br>**92.8/1.2e-24 (6.4e-23)**<br><br>**47.9/2.7e-11 (3.1e-27)**<br><br>**24.3/5.1e-09 (6.4e-23)** |
| PF01569.13 :<br>PAP2<br>(type 2 phosphatidic acid phosphatase)<br><br>Gathering score : 8.3<br>Alignment length: 261<br>HMM length: 177 | TM,200-261 | 129-143,<br>156-172<br><br>ref.[2] | **5. XP_418136.2 (1153 AA)** | Similar to Aoc2 protein, *Gallus gallus* | **859-1000** | **54.1/5.3e-13 (1e-06)** |
| PF01105.15 :<br>EMP24_GP25L<br>(Endoplasmic reticulum and golgi apparatus trafficking proteins)<br><br>Gathering score : -16<br>Alignment length: 346<br>HMM length: 167 | TM,315-346 | 142-162<br><br>ref.[3] | **6. CAN62859.1 (1181 AA)** | hypothetical protein, *Vitis vinifera* | **1018-1173** | **56.7/9e-14 (5.1e-11)** |
| PF04387.6 :<br>PTPLA<br>(protein tyrosine phosphatase-like protein)<br><br>Gathering score : 25<br>Alignment length: 177<br>HMM length: 168 | TM,98-177 | 89-106,<br>138-155<br><br>refs.[4,5] | **7. EAY72555.1 (646 AA)**<br>EAZ10566.1(336AA)+ BAH90915.1(342AA) +*EEC69961.1(198AA)* | hypothetical protein OsI_000402, *Oryza sativa* | **523-646**<br>*63-194* | **-19.5/1.9e-05 (1.6e-15)**<br>*26.2/6.1e-09 (1.6e-20)* |
| PF01299.9 :<br>Lamp<br>(Lysosome-associated membrane glycoprotein)<br><br>Gathering score : -87<br>Alignment length: 369<br>HMM length: 340 | TM,328-369 | 304-327<br><br>ref.[6] | **8. XP_487300.2 (321 AA)**<br>*NP_001139351.1(336 AA)*<br><br>**9. XP_916963.1 (321 AA)**<br>*NP_001139351.1(336 AA)* | hypothetical protein, *Mus musculus*<br><br>hypothetical protein, *Mus musculus* | **50-280**<br>*65-295*<br><br>**50-280**<br>*65-295* | **-71.3/1.4e-04 (3.3e-11)**<br><br>**-71.3/1.4e-04 (3.3e-11)** |

| | | | | | | |
|---|---|---|---|---|---|---|
| PF02416.8 :<br>MttA_Hcf106<br>(sec-independent translocation mechanism protein)<br><br>Gathering score : 7<br>Alignment length: 83<br>HMM length: 74 | TM,1-22 | 1-19<br><br>refs.[7,8] | **10. ZP_00374359.1 (256 AA)** | RNA polymerase sigma factor RpoD, Wolbachia endosymbiont of *Drosophila ananassae* | **204-255** | **47.6/5e-11 (9.2e-12)** |
| | | | **11. ZP_02966160.1 (244 AA)**<br>*ZP_03628932.1(244AA)* | phosphatidylglycerophosphatase A, *bacterium Ellin514* | **1-60** | **15.6/1.4e-04 (6.5e-06)** |
| F00672.17 :<br>HAMP<br>(cytoplasmic helical linker domain)<br><br>Gathering score : 19.8<br>Alignment length: 106<br>HMM length: 79 | TM,1-23 | 1-15<br><br>ref.[9] | **12. ZP_02846008.1 (755 AA)**<br>*YP_003010496.1(755 AA)* | Transcriptional regulator AraC family, *Paenibacillus sp. JDR-2* | **297-360** | **37.8/4.4e-08 (6.3e-07)** |
| | | | **13. ZP_01574605.1 (760 AA)**<br>*YP_002504510.1(760 AA)* | Transcriptional regulator AraC family, *Clostridium cellulolyticum H10* | **300-364** | **30.8/5.7e-06 (1.9e-05)** |
| | | | **14. ZP_02847254.1 (788 AA)**<br>*YP_003012870.1(788 AA)* | Transcriptional regulator AraC family, *Paenibacillus sp. JDR-2* | **304-371** | **28.2/3.4e-05 (3.5e02)** |
| | | | **15. ZP_03039254.1 (756 AA)**<br>*YP_003244876.1(756 AA)* | helix-turn-helix domain containing protein AraC type, *Geobacillus sp. Y412MC10* | **297-360** | **26.7/9.7e-05 (9.7e-04)** |
| PF07127.3 :<br>Nodulin_late<br>(plant specific late nodulin)<br><br>Gathering score : 25<br>Alignment length: 69<br>HMM length: 67 | SP,1-28 | 1-25<br><br>ref.[10] | **16. ABD33411.1 (175 AA)** | Terpenoid cyclase/protein prenyltransferase alpha-alpha toroid; Terpenoid synthase; Late nodulin, *Medicago truncatula* | **1-41** | **35.4/2.3e-07 (7.7e-11)** |
| PF07172.3 :<br>GRP<br>(plant glycine rich proteins)<br><br>Gathering score : 17.2<br>Alignment length: 145<br>HMM length: 134 | SP,1-29 | 1-49<br><br>ref.[11] | **17. CAL51691.1 (693 AA)** | Putative RNA helicase (ISS), *Ostreococcus tauril* | **582-692** | **1.8/5e-05 (NA*)** |

In the first column, we list selected Pfam domains with their accession, identifier, description and their gathering score (as in Pfam release 23) that have TM and/or SP regions included into the model. We also provide alignment length and the HMM length. The latter might be considerably shorter than the former as a result of hmmbuild defaults in HMMER2.

The region in the domain alignment that includes the predicted SP/TM segments (together with interlinking loops as described in Methods) is provided in the second column. We searched for experimental proof of these predictions in the literature and the corresponding references and the positional ranges for the

respective SP/TM segments (with respect to the HMM but not the alignment) are given in the third column.

The next two columns provide running number, accession (in bold), sequence length (in bold), description and taxonomic origin of sequences that were found as false-positive hits of the respective HMMs when using HMMER2 in the global-mode search. The penultimate column shows the range of the hit in the subject sequence (at the domain side, the hit was always over the full length of the HMM, in bold font). The last column provides score and E-value for HMMER2 and (in parentheses) the E-value with HMMER3/Pfam release 24 as by the web server http://pfam.sanger.ac.uk (October 2009, all values in bold font). * denotes that the problematic domain annotation is not found by HMMER3/Pfam release 24 in the local search mode.

During the revision of this manuscript, several sequence entries have been updated either fully or partially. In these cases, the old sequence entries have been complemented by new accession numbers (in italic). Any subsequent changes affecting computational results (with regard to their corresponding positional changes, raw scores and E-values) are also provided in italic font if applicable.

Additional material such as hmmpfam outputs and alignments are available at the associated BII WWW site for this work.

References


1. Watanabe R, Murakami Y, Marmor MD, Inoue N, Maeda Y, Hino J, Kangawa K, Julius M, Kinoshita T (2000) Initial enzyme for glycosylphosphatidylinositol biosynthesis requires PIG-P and is regulated by DPM2. EMBO J 19: 4402-4411.

2. Sun L, Gu S, Sun Y, Zheng D, Wu Q, Li X, Dai J, Dai J, Ji C, Xie Y, Mao Y (2005) Cloning and characterization of a novel human phosphatidic acid phosphatase type 2, PAP2d, with two different transcripts PAP2d_v1 and PAP2d_v2. Mol Cell Biochem 272: 91-96.

3. Ciufo LF, Boyd A (2000) Identification of a lumenal sequence specifying the assembly of Emp24p into p24 complexes in the yeast secretory pathway. J Biol Chem 275: 8382-8388.

4. Kihara A, Sakuraba H, Ikeda M, Denpoh A, Igarashi Y (2008) Membrane topology and essential amino acid residues of Phs1, a 3-hydroxyacyl-CoA dehydratase involved in very long-chain fatty acid elongation. J Biol Chem 283: 11199-11209.

5. Uwanogho DA, Hardcastle Z, Balogh P, Mirza G, Thornburg KL, Ragoussis J, Sharpe PT (1999) Molecular cloning, chromosomal mapping, and developmental expression of a novel protein tyrosine phosphatase-like gene. Genomics 62: 406-416.

6. Fukuda M (1991) Lysosomal membrane glycoproteins. Structure, biosynthesis, and intracellular trafficking. J Biol Chem 266: 21327-21330.

7. Settles AM, Yonetani A, Baron A, Bush DR, Cline K, Martienssen R (1997) Sec-independent protein translocation by the maize Hcf106 protein. Science 278: 1467-1470.

8. Weiner JH, Bilous PT, Shaw GM, Lubitz SP, Frost L, Thomas GH, Cole JA, Turner RJ (1998) A novel and ubiquitous system for membrane targeting and secretion of cofactor-containing proteins. Cell 93: 93-101.

9. Aravind L, Ponting CP (1999) The cytoplasmic helical linker domain of receptor histidine kinase and methyl-accepting proteins is common to many prokaryotic signalling proteins. FEMS Microbiol Lett 176: 111-116.

10. Scheres B, van EF, van der KE, van de WC, van KA, Bisseling T (1990) Sequential induction of nodulin gene expression in the developing pea nodule. Plant Cell 2: 687-700.

11. de Oliveira DE, Seurinck J, Inze D, Van MM, Botterman J (1990) Differential expression of five Arabidopsis genes encoding glycine-rich proteins. Plant Cell 2: 427-436.
