# Supplementary material for: More Than 1,001 Problems with Protein Domain Databases: Transmembrane Regions, Signal Peptides and the Issue of Sequence Homology
Source: PLoS Comput Biol. 2010 Jul 29;6(7):e1000867. doi: 10.1371/journal.pcbi.1000867 (PMC2912341; doi:10.1371/journal.pcbi.1000867)
Supplement: Table S3 — Summary of selected false-negative sequence hits with problematic domain annotations (global-mode search). (0.05 MB PDF) [file pcbi.1000867.s006.pdf]

Supplementary file: Table S3. Summary of selected false-negatives sequence hits with respect to problematic domains (Global-mode search)

**Domain Name**	**Type, predicted region of alignment**	**Validated TM helices/ SP of model, reference**	**Sequence accession no. (No. of AA)**	**Sequence Description/ Taxonomy**	**Raw score/ E-value of FN hits with HMMER2**	**Raw score/ E-value of FN hits less SP/TM segments with HMMER2**
PF08510.4 :<br>PIG-P<br>(phosphatidylinositol N-acetylGlucosaminyl transferase subunit P)<br><br>Gathering score : -11.4<br>Alignment length: 208<br>HMM length: 153	TM,1-91	8-24,<br>44-67<br><br>ref.[1]	1. EDL76721.1<br>(55 AA, fragment)	phosphatidylinositol glycan, class P, isoform CRA_b, *Rattus norvegicus*	-25.9/3.4e-4	67.9/4.1e-9
PF01569.13 :<br>PAP2<br>(type 2 phosphatidic acid phosphatase)<br><br>Gathering score : 8.3<br>Alignment length: 261<br>HMM length: 177	TM,200-261	129-143,<br>156-172<br><br>ref.[2]	2. NP_001096030.1<br>(176 AA, complete)	phosphatidate phosphatase PPAPDC1B isoform 3, *Homo sapiens*	-1.0/6.7e-3	52.1/2.2e-12
PF04387.6 :<br>PTPLA<br>(protein tyrosine phosphatase-like protein)<br><br>Gathering score : 25<br>Alignment length: 177<br>HMM length: 168	TM,98-177	89-106,<br>138-155<br><br>refs.[3,4]	3. NP_001012396.2<br>(165 AA, complete)<br><br>4. CAI46276.1<br>(136AA, complete)	protein-tyrosine phosphatase-like member A isoform 2, *Mus musculus*<br><br>protein tyrosine phosphatase-like, member a, splice variant PTPLAd5, *Canis familiaris*	-30.4/1.3e-4<br><br>-65.3/5.9e-2	32.6/2.0e-09<br><br>28.0/4.5e-09
F00672.17 :<br>HAMP<br>(cytoplasmic helical linker domain)<br><br>Gathering score : 19.8<br>Alignment length: 106<br>HMM length: 79	TM,1-23	1-15<br><br>ref.[5]	5. NP_661555.1<br>(399AA, complete)	sensor histidine kinase, *Chlorobium tepidum TLS*	9.7/2.2e-1	45.3/2.4e-10
PF00690.18 :<br>Cation_ATPase_N<br>(Cation transporter/ATPase, N-terminus)<br><br>Gathering score : 18.9<br>Alignment length: 107<br>HMM length: 87	TM, 85-105	TM,66-87<br><br>ref.[6]	6. XP_001868895.1<br>(814AA, complete)	calcium-transporting ATPase sarcoplasmic/endoplasmic reticulum type, *Culex quinquefasciatus*	7.1/8.1e-4	26.9/7.9e-06
PF01544.10 :<br>CorA<br>(CorA-like Mg2+ transporter protein)<br><br>Gathering score : -61.3<br>Alignment length: 550<br>HMM length: 407	TM, 482-544	TM,341-407<br><br>ref.[7]	7. AAO72700.1<br>(158AA, fragment)	putative Mg transporter, *Oryza sativa Japonica Group*	-61.8/3.8e-3	17.3/4.8e-07

PF00558.11 :<br>Vpu<br>(Vpu protein)<br><br>Gathering score : -9.5<br>Alignment length: 101<br>HMM length: 96	TM, 12-37	TM,6-28<br><br>ref.[8]	8. P08808<br>(34AA, fragment)	Protein Vpu, *Human immunodeficiency virus type 1 (WMJ2 ISOLATE)*	-16.0/1.3e-2	36.6/9.8e-08
PF07365.4 :<br>Toxin_8<br>(Alpha conotoxin precursor)<br><br>Gathering score : 25<br>Alignment length: 71<br>HMM length: 66	SP, 1-21	SP,1-21<br><br>ref.[9]	9. AAZ85382.1<br>(38AA, fragment)	TeA21P, *Conus textile*	16.1/1.5e-2	45.0/2.9e-10
			10. Q1L777.1<br>(38AA, fragment)	Alpha-conotoxin PeIA, *Conus pergrandis*	14.0/2.6e-2	42.9/1.2e-09
			11. ABD33869.1<br>(38AA, fragment)	alpha conotoxin Mr1.1, *Conus marmoreus*	12.8/3.6e-2	41.7/2.8e-09
			12. ABD33862.1<br>(41AA, fragment)	alpha conotoxin Qc1.5, *Conus quercinus*	10.7/6.1e-2	39.6/1.2e-08
			13. ABD48795.1<br>(45AA, fragment)	conotoxin Lp1.10, *Conus leopardus*	15.0/2.1e-2	37.2/6. 5e-08
			14. ABD33857.1<br>(41AA, fragment)	alpha conotoxin Qca-L-1, *Conus quercinus*	8.1/0.12	37.0/7.3e-08
			15. P69658.1<br>(40AA, fragment)	Alpha-conotoxin PIA, *Conus purpurascens*	4.6/0.3	33.5/8.3e-07
			16. AAZ85381.1	LeD2P, *Conus litteratus*	1.5/0.67	30.4/7.1e-06
			17. ABD33863.1<br>(38AA, fragment)	alpha conotoxin Qc1.6, *Conus quercinus*	0.8/0.79	29.7/1.2e-05
			18. ABD33860.1<br>(40AA, fragment)	alpha conotoxin Qc1.4a, *Conus quercinus*	0.2/0.93	29.1/1.8e-05
			19. ABD33867.1<br>(53AA, fragment)	alpha conotoxin Ac4.3b, *Conus achatinus*	-0.8/1.2	28.1/3.5e-05
			20. ABD33865.1<br>(41AA, fragment)	alpha conotoxin Ac4.2, *Conus achatinus*	-1.3/1.4	27.6/5.0e-05
			21. ABD33870.1<br>(41AA, fragment)	alpha conotoxin Pu1.2, *Conus pulicarius*	-1.9/1.6	27.0/7.5e-05
			22. AAZ85380.1<br>(40AA, fragment)	LiC22P, *Conus lividus*	-3.0/2.1	25.9/1.6e-4
PF00482.11 :<br>GSPII_F<br>(Bacterial type II secretion system protein F domain)<br><br>Gathering score : 25<br>Alignment length: 185<br>HMM length: 136	TM, 166-184	TM,118-136<br><br>ref. [10]	23. ZP_03270751.1<br>(163AA, fragment)	type II secretion system, subunit F/type IV pilus assembly protein TapC/PilC, *Burkholderia sp. H160*	0.1/7.6e-1	26.9/8.5e-05
PF04901.5 :<br>RAMP<br>(Receptor activity	TM, 85-106	TM, 85-106<br><br>ref. [11]	24. NP_848488.2<br>(103AA, complete)	receptor activity-modifying protein 1 isoform 2, *Mus*	-24.9/3.4e-2	5.4/7.3e-05

modifying family)<br><br>Gathering score : 25<br>Alignment length: 115<br>HMM length: 115				*musculus*		

In the first column, we list selected Pfam domains with their accession, identifier, description and their gathering score (as in Pfam release 23) that have TM and/or SP regions included into the model. We also provide alignment length and the HMM length. The latter might be considerably shorter than the former as a result of hmmbuild defaults in HMMER2.

The region in the domain alignment that includes the predicted SP/TM segments (together with interlinking loops as described in Methods) is provided in the second column. We searched for experimental proof of these predictions in the literature and the corresponding references and the positional ranges for the respective SP/TM segments (with respect to the HMM but not the alignment) are given in the third column.

The next two columns provide running number, accession, sequence length, description and taxonomic origin of sequences that were found as false-negative hits of the respective HMMs when using HMMER2 in the global-mode search. The penultimate column provides their scores and E-values based on HMMER2. The last column gives the recomputed scores and E-values of the false-negative hits without their respective SP/TM segments.

Additional material such as hmmpfam outputs and alignments are available at the associated BII WWW site for this work.

References

1. Watanabe R, Murakami Y, Marmor MD, Inoue N, Maeda Y, Hino J, Kangawa K, Julius M, Kinoshita T (2000) Initial enzyme for glycosylphosphatidylinositol biosynthesis requires PIG-P and is regulated by DPM2. EMBO J 19: 4402-4411.

2. Sun L, Gu S, Sun Y, Zheng D, Wu Q, Li X, Dai J, Dai J, Ji C, Xie Y, Mao Y (2005) Cloning and characterization of a novel human phosphatidic acid phosphatase type 2, PAP2d, with two different transcripts PAP2d_v1 and PAP2d_v2. Mol Cell Biochem 272: 91-96.

3. Kihara A, Sakuraba H, Ikeda M, Denpoh A, Igarashi Y (2008) Membrane topology and essential amino acid residues of Phs1, a 3-hydroxyacyl-CoA dehydratase involved in very long-chain fatty acid elongation. J Biol Chem 283: 11199-11209.

4. Uwanogho DA, Hardcastle Z, Balogh P, Mirza G, Thornburg KL, Ragoussis J, Sharpe PT (1999) Molecular cloning, chromosomal mapping, and developmental expression of a novel protein tyrosine phosphatase-like gene. Genomics 62: 406-416.

5. Aravind L, Ponting CP (1999) The cytoplasmic helical linker domain of receptor histidine kinase and methyl-accepting proteins is common to many prokaryotic signalling proteins. FEMS Microbiol Lett 176: 111-116.

6. Xu C, Rice WJ, He W, Stokes DL (2002) A structural model for the catalytic cycle of Ca(2+)-ATPase. J Mol Biol 316: 201-211.

7. Lunin VV, Dobrovetsky E, Khutoreskaya G, Zhang R, Joachimiak A, Doyle DA, Bochkarev A, Maguire ME, Edwards AM, Koth CM (2006) Crystal structure of the CorA Mg2+ transporter. Nature 440: 833-837.

8. Montal M (2003) Structure-function correlates of Vpu, a membrane protein of HIV-1. FEBS Lett 552: 47-53.

9. Chi SW, Kim DH, Olivera BM, McIntosh JM, Han KH (2006) Solution conformation of a neuronal nicotinic acetylcholine receptor antagonist alpha-conotoxin OmIA that discriminates alpha3 vs. alpha6 nAChR subtypes. Biochem Biophys Res Commun 345: 248-254.

10. Abendroth J, Mitchell DD, Korotkov KV, Johnson TL, Kreger A, Sandkvist M, Hol WG (2009) The three-dimensional structure of the cytoplasmic domains of EpsF from the type 2 secretion system of Vibrio cholerae. J Struct Biol 166: 303-315.

11. Kusano S, Kukimoto-Niino M, Akasaka R, Toyama M, Terada T, Shirouzu M, Shindo T, Yokoyama S (2008) Crystal structure of the human receptor activity-modifying protein 1 extracellular domain. Protein Sci 17: 1907-1914.
